# Cathodoluminescence Analysis of Defects and Grain Boundaries in Zn_3_P_2_ Thin Films Grown on Graphene by MOVPE and MBE

**DOI:** 10.1021/acsmaterialsau.6c00120

**Published:** 2026-07-28

**Authors:** Thomas Hagger, Mohammadreza Hassanzadeh, Aidas Urbonavicius, Ahmed El Alouani, Victor Boureau, Gulnaz Ganeeva, Nico Kawashima, Raphael Lemerle, Kamil Artur Wodzislawski, Sebastian Lehmann, Kimberly A. Dick, Silvana Botti, Adrien Michon, Anna Fontcuberta i Morral, Simon Escobar Steinvall

**Affiliations:** † Laboratory of Semiconductor Materials, Institute of Materials, School of Engineering, École Polytechnique Fédérale de Lausanne (EPFL), 1015 Lausanne, Switzerland; ‡ Centre for Analysis and Synthesis and NanoLund, 5193 Lund University, Box 124, 221 00 Lund, Sweden; § Université Côte d’Azur, CNRS, CRHEA, Rue Bernard Grégory, 06560 Valbonne, France; ∥ Interdisciplinary Center for Electron Microscopy (CIME), EPFL, 1015 Lausanne, Switzerland; ⊥ Research Center Future Energy Materials and Systems of the University Alliance Ruhr and ICAMS, Ruhr University Bochum, Universitätsstraße 150, D-44801 Bochum, Germany; # Institut für Festkörpertheorie und-optik, Friedrich-Schiller-Universität Jena, Max-Wien-Platz 1, 07743 Jena, Germany

**Keywords:** Zn_3_P_2_, quasi-van der Waals epitaxy, graphene, cathodoluminescence, antiphase boundaries, grain boundaries

## Abstract

Zn_3_P_2_ is a promising earth-abundant absorber for thin-film photovoltaics, yet its development is hindered by the lack of lattice-matched substrates, its incompatible thermal expansion coefficient, and a complex defect landscape. Here, we demonstrate the quasi-van der Waals epitaxy of Zn_3_P_2_ on graphene by metal–organic vapor phase epitaxy (MOVPE) and demonstrate a clear spatial correlation between antiphase boundary density and local variations in the optical emission using correlative electron microscopy and cathodoluminescence (CL). Moreover, CL measurements show that grain boundaries act as efficient non-radiative recombination centers, with an effective recovery length extending several micrometres into the grains, indicating that grain boundaries can significantly influence carrier transport and recombination in Zn_3_P_2_. Further comparison with molecular beam epitaxy grown films reveals the suppression of strain-related sub-bandgap emission in MOVPE-grown material. Overall, quasi-van der Waals epitaxy of Zn_3_P_2_ by MOVPE resulted in larger grains and improved material quality. These results establish clear relationships between extended defects and the local optical response in Zn_3_P_2_, while highlighting grain-size control as a key strategy for improving earth-abundant photovoltaic absorbers.

## Graphical abstract

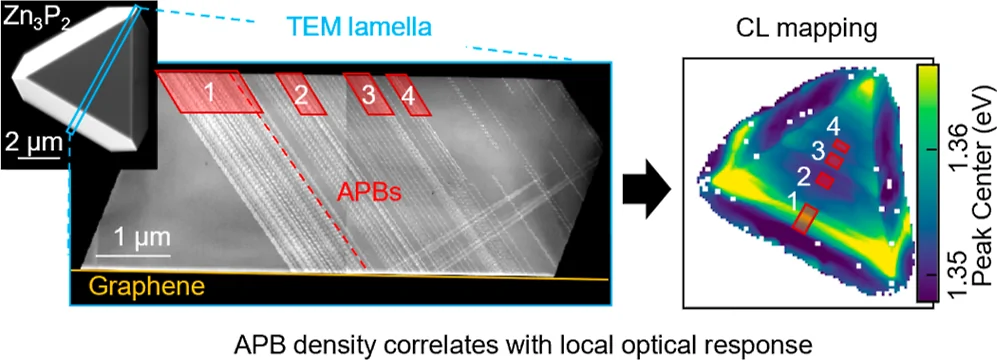

## Introduction

Established thin-film photovoltaic technologies such as CdTe and CIGS rely on elements that are either scarce or toxic, which may limit their long-term scalability.^1–6^ Emerging alternatives such as perovskites and kesterites avoid some of these concerns but face other challenges, including stability, toxicity issues or complex defect chemistry.^7–9^ This motivates the exploration of additional earth-abundant absorber materials for sustainable thin-film photovoltaic technologies.

One promising candidate is Zn_3_P_2_, a compound semiconductor composed of earth-abundant elements and exhibiting favorable optoelectronic properties for photovoltaic applications. It possesses a direct bandgap of approximately 1.5 eV, close to the optimum for single-junction solar cells, and a high absorption coefficient exceeding 10^4^ cm^−1^ in the visible spectral range.^10–13^ In addition, long minority carrier diffusion lengths in the range of 5–7 μm have been reported.^14,15^ Early work on this material demonstrated a record conversion efficiency of nearly 6% in 1981.^16^ Despite a theoretical detailed balance conversion efficiency limit close to 32%,^17^ this efficiency has not been improved since, suggesting that fundamental material challenges remain.

One major challenge arises from the large lattice constants and thermal expansion coefficient of Zn_3_P_2_ relative to commonly used substrates, which complicates the growth of high-quality monocrystalline thin films.^18–20^ High crystalline quality has been achieved using InP substrates, where monocrystalline films up to approximately 1 μm thickness have been reported,^21^ and more recently, further improvements have been achieved using selective area epitaxy, where growth is initiated in nanoscale mask openings that allow strain relaxation before lateral overgrowth forms a continuous film.^22^ However, these substrates currently rely on indium, a scarce element, which conflicts with the goal of developing a fully earth-abundant thin-film photovoltaic absorber.

Another challenge arises from the complex defect landscape of Zn_3_P_2_. The material is typically intrinsically p-type, and achieving stable n-type doping has proven difficult.^11,23^ First-principles calculations attribute this behavior to the low formation energies of several intrinsic defects, which promote strong self-compensation and complicate compositional control.^24,25^ In addition to point defects, structural disorder such as strain fields and rotational domains has been reported in Zn_3_P_2_ thin films and can lead to spatial variations in optical response.^26,27^ In many polycrystalline photovoltaic absorbers, such as Si, CdTe, CIGS, kesterites and hybrid perovskites, grain boundaries can strongly influence carrier recombination and transport.^28–36^ However, the influence of grain boundaries on the optoelectronic properties of Zn_3_P_2_ remains poorly understood. Early work suggested that grain boundaries may not strongly limit device performance,^16^ whereas later studies indicated that they can deteriorate electronic and optoelectronic properties.^37^ Clarifying the role of extended defects such as grain boundaries is therefore important for understanding recombination processes and guiding the development of improved growth strategies.

Quasi–van der Waals epitaxy has emerged as a promising strategy by growing three-dimensional materials on two-dimensional substrates such as graphene.^38,39^ In this growth mode, the absence of strong covalent bonding at the interface relaxes lattice-matching constraints and can reduce strain during epitaxial growth. In addition, the weak interfacial bonding enables relatively straightforward exfoliation of the grown film and reuse of the substrate, which can reduce material consumption and improve the sustainability of the growth process.^40^ Using this approach, Paul et al. demonstrated the growth of Zn_3_P_2_ on graphene by molecular beam epitaxy (MBE).^41^ The resulting films exhibit a well-defined out-of-plane orientation but retain rotational freedom in the plane, leading to inherently polycrystalline thin films. Subsequent work showed that the grain size was limited to approximately 2 μm and strongly correlated with the quality of the graphene substrate, whereas variations in MBE growth parameters did not significantly increase the grain size.^27^

These observations suggest that the growth kinetics of MBE may inherently favor high nucleation densities, limiting the achievable grain size. This raises the question of whether alternative growth techniques could enable improved control over nucleation and grain size. A recent review on transition-metal dichalcogenide (TMD) growth highlighted that the grain size of two-dimensional materials can depend strongly on the growth technique.^42^ In particular, MBE, which is a ballistic growth method, often results in higher nucleation densities and therefore smaller grain sizes compared to chemical vapor deposition (CVD) or metal–organic vapor phase epitaxy (MOVPE). These latter techniques typically operate at higher growth temperatures and involve surface-mediated precursor decomposition, which can increase surface diffusion lengths and favor the lateral growth of larger grains. This suggests that MOVPE may provide a promising route to achieve larger Zn_3_P_2_ grains on graphene.

Despite recent progress in Zn_3_P_2_ photovoltaics, the influence of intrinsic defects, stoichiometry, extended defects and interfaces on carrier recombination and transport remains incompletely understood. Well-defined material systems, therefore, provide an important model platform for disentangling intrinsic material properties from microstructural effects and establishing structure–property relationships. Such understanding is essential for guiding the development of scalable photovoltaic architectures, whether through improved growth strategies, grain-boundary passivation, postgrowth treatments, optimization of charge-selective contacts or other approaches to microstructure engineering.

In this work, we demonstrate the growth of Zn_3_P_2_ on graphene by MOVPE and investigate the relationship between structural defects and local optical response using combined electron microscopy and hyperspectral cathodoluminescence. By correlating antiphase boundary (APB) density and grain boundaries with spatially resolved luminescence, we provide insight into the defect-related recombination behavior governing polycrystalline Zn_3_P_2_ and evaluate the potential of MOVPE-grown material for photovoltaic applications.

## Results and Discussion

### MOVPE Growth

Zn_3_P_2_ was successfully grown by MOVPE on epitaxial graphene supported on semi-insulating 4H–SiC substrates. The graphene was prepared by hydrogen-assisted chemical vapor deposition (H–CVD). In particular, the MOVPE growth results in substantially larger grains than previously reported for MBE-grown Zn_3_P_2_ on graphene.^27,41^ Representative scanning electron microscopy (SEM) images of two magnifications obtained under different pre-growth annealing and growth conditions are shown in Figure 1 to showcase changes in grain size, morphology and nucleation density. The images were acquired near the center of the substrate to minimize edge-related variations in growth conditions. While some spatial inhomogeneity was observed across the 1 × 1 cm^2^ chips, the selected regions are representative of the dominant growth morphology.

**Figure 1:**
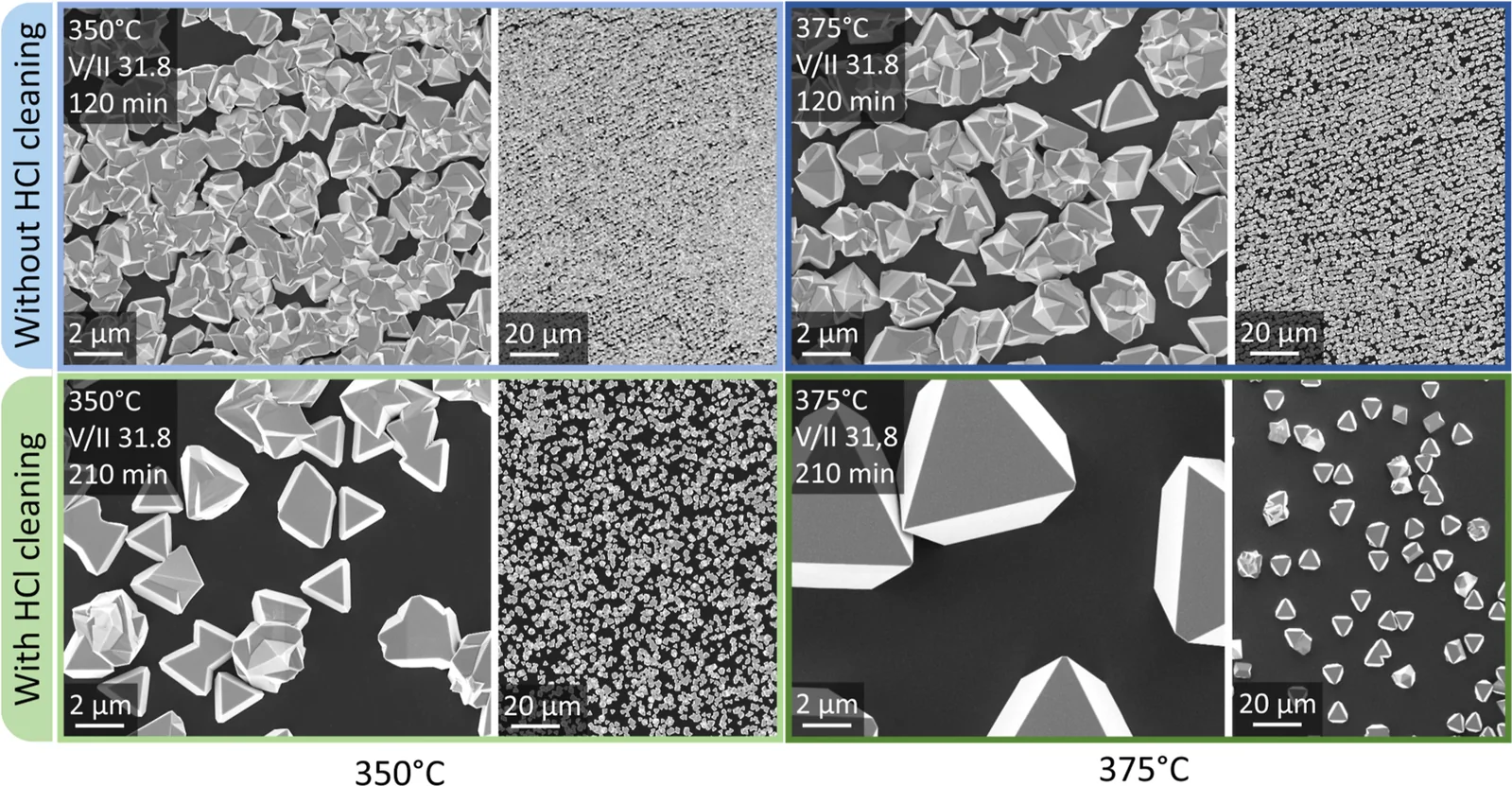
Two magnifications of top-view SEM images of Zn_3_P_2_ grown by MOVPE on epitaxial graphene/SiC substrates under different pre-growth annealing and growth conditions. The top row corresponds to thermal annealing + PH_3_ treatment of the graphene prior to Zn_3_P_2_ growth, whereas the bottom row corresponds to thermal annealing + PH_3_ + HCl_(g)_ treatment. Growth conditions and times are indicated in the upper left corner of each image. Scale bars: 2 and 20 μm.

The films consist of triangular grains, consistent with the intrinsic growth habit of Zn_3_P_2_ previously reported for growth on graphene.^27,41^ The grains exhibit a preferential top facet of a {101} plane while maintaining rotational freedom in-plane, resulting in polycrystalline films. A pronounced tendency for nucleation along linear features is visible in several samples. These lines correspond to the terraces of the underlying SiC substrate beneath the graphene, indicating that the substrate morphology influences the nucleation of Zn_3_P_2_ islands. Similar morphology-driven nucleation behavior has also been observed in other van der Waals epitaxy systems.^27^

A strong influence of the pre-growth surface preparation on the resulting morphology is observed. Substrates treated with thermal annealing + phosphine (PH_3_) prior to the growth show a higher density of misoriented or parasitic grains compared to the ones with an additional gaseous hydrochloric acid (HCl_(g)_) flow during the thermal annealing. Increasing the growth temperature improves morphology, resulting in a greater number of better-defined triangular grains, especially when HCl_(g)_ cleaning is included. Raman spectroscopy performed before and after growth (see Figure S1 in the Supporting Information) confirms that the graphene layer remains intact, indicating that the HCl_(g)_ treatment cleans the graphene surface without introducing detectable damage.

This behavior indicates that surface contamination plays a key role in the nucleation of Zn_3_P_2_ on graphene. The observed reduction in nucleation density with in situ HCl_(g)_ treatment is therefore consistent with the removal of surface contaminants, resulting in a cleaner and more homogeneous graphene surface. As a consequence, a lower nucleation density enables the development of larger crystal grains. Further improvements in morphology are achieved by increasing the growth temperature in combination with the HCl_(g)_ treatment, yielding substantially larger and more well-defined triangular grains. This behavior can be attributed to enhanced adatom surface diffusion at elevated temperatures, and changes in the decomposition kinetics and surface transport of the metal–organic precursors and their intermediate species, which together influence the effective supply and incorporation of reactive species. Similar trends are observed across different graphene substrate qualities (see Figures S2–S4), indicating that this behavior is robust.

Because Zn_3_P_2_ growth on graphene results in polycrystalline films due to the rotational freedom of the grains, increasing the grain size is particularly important for potential device applications, as it reduces the density of grain boundaries that may negatively affect electronic and optoelectronic properties. In polycrystalline semiconductors, grain boundaries are known to introduce recombination-active defect states and electrostatic potential barriers that can modify carrier transport and, in some cases, act as electrical shunts that increase the dark current.^28,29^ Consistent with this behavior, early studies on thin-film Zn_3_P_2_ have reported that surface states and grain boundaries can strongly influence the photoconductivity response and carrier dynamics.^37^

Previously reported MBE growth of Zn_3_P_2_ on graphene yields grain sizes of up to approximately 2 μm. In contrast, MOVPE produces grains approaching 10 μm, which does not represent the maximum size, as full coalescence has not yet been achieved.^27,41^ This substantial increase in grain size indicates a significantly lower nucleation density during MOVPE growth. Such behavior is consistent with observations in other van der Waals epitaxy systems, such as transition-metal dichalcogenides, where higher growth temperatures and different growth kinetics result in lower nucleation densities and, consequently, larger crystal grains.^42,43^

The reduced nucleation density observed in MOVPE growth compared to MBE can be attributed to differences in growth kinetics. MOVPE operates at a growth temperature approximately 150 °C higher and involves precursor decomposition at the substrate surface, thereby promoting enhanced adatom mobility and changes in the transport and reactivity of precursor-derived species prior to incorporation. In contrast, MBE is a ballistic growth method in which species arrive with limited surface diffusion.^44^ Since surface diffusion follows an Arrhenius-type behavior, even moderate increases in temperature lead to an exponential increase in diffusion length.^45^ The resulting enhanced mobility reduces the probability of new nucleation events and instead favors the lateral expansion of existing islands. Consequently, fewer nuclei form, provided that the supersaturation remains comparable, allowing grains to grow to significantly larger sizes. Reducing precursor supply could, in principle, achieve a similar effect, but is constrained by the already low growth rates of Zn_3_P_2_ in MOVPE. Although a complete understanding of the influence of the individual growth parameters requires further systematic investigation, the present results demonstrate that MOVPE provides a promising route to substantially increase the grain size of Zn_3_P_2_ grown on graphene, while graphene remains stable under the pre-growth annealing conditions required to achieve these improvements.

### Structural Defects in MOVPE Zn_3_P_2_

Aberration-corrected scanning transmission electron microscopy (STEM) was performed on a single large Zn_3_P_2_ grain grown at 375 °C and a precursor flux ratio V/II = 41.7, as shown in Figure 2. Precession 4D-STEM was used on the grain cross-section observed along the [100] orientation, as indicated in the SEM image in Figure 2a, for large field-of-view analysis of the crystal structure. This technique collects the diffraction patterns at each pixel of the map, allowing to confirm the single-crystalline nature of the grain. A typical diffraction pattern is shown in Figure 2b.

**Figure 2:**
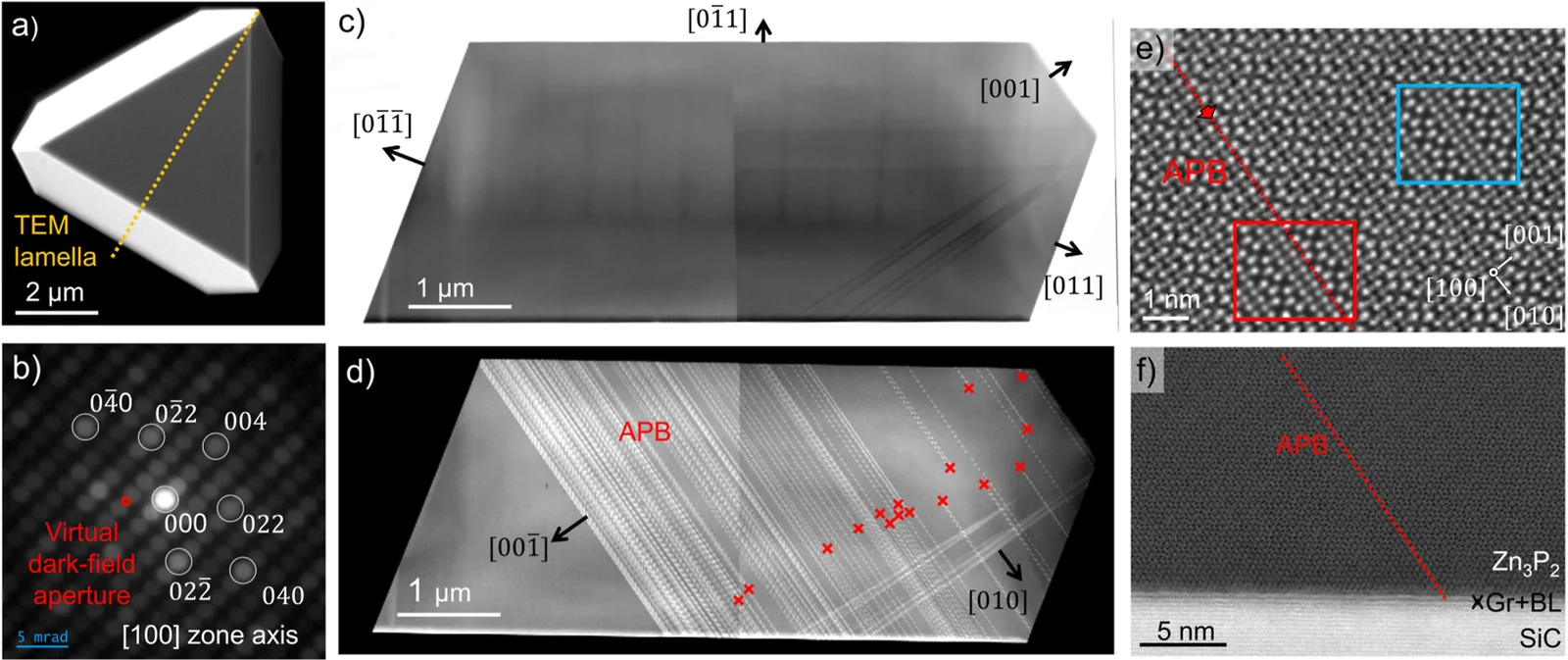
(a) SEM image of a Zn_3_P_2_ crystal indicating the location of the TEM lamella extraction. (b–d) Precession 4D-STEM results. (b) Is the electron diffraction pattern observed in the Zn_3_P_2_ grain revealing the tetragonal crystal observed in [100] zone axis with out-of-plane orientation [011]. The red square indicates the virtual aperture used to generate the VDF image. (c) and (d) are the VBF and VDF images, respectively, and specific crystal orientations are annotated. The VDF detector was adjusted to generate a high contrast from the APBs. Most of these planar defects run across the grain thickness, while red crosses indicate APB termination. (e) Atomic-resolution HAADF STEM image of Zn_3_P_2_, where an APB on (001) is observed along the red dashed line with a displacement vector 1/2[010] indicated with the arrow. Red and blue insets are the image simulation with and without APB, respectively. (f) Atomic-resolution BF-STEM image of the interface between Zn_3_P_2_, graphene, buffer layer and SiC. An APB is indicated by the red dashed line.

The virtual bright-field (VBF) image (Figure 2c) is built using the intensity of the transmitted beam, and the facets are indexed using the reflections observed in the diffraction pattern. The diffraction pattern indicates that the facets correspond to planes with surface normals along the ⟨011⟩ family, i.e., {011} facets. This is in agreement with the literature, where it has been shown that ⟨011⟩ facets are the lowest energy and most stable facets for Zn_3_P_2_.^22,46^

A virtual dark-field (VDF) image (Figure 2d), built from the intensity circled by the virtual aperture indicated in the diffraction pattern (Figure 2b), reveals a network of extended planar defects running across the crystal.^47^ These defects appear as parallel lines with well-defined orientations and exhibit a spatially varying density across the lamella. In some regions, the defects are densely packed and continuous over micrometre length scales, while in others they are sparsely distributed and locally interrupted. Such discontinuities are highlighted by red crosses in Figure 2d.

Atomic resolution high-angle annular dark-field (HAADF) STEM imaging (Figure 2e) identifies these planar defects as antiphase boundaries (APBs). An APB is defined as a planar defect separating two regions of identical crystallographic orientation that are shifted with respect to each other by a translation vector of the ordered lattice, resulting in a phase shift of the atomic sublattice.^48^ In contrast to stacking faults, which arise from a local disruption of the stacking sequence, APBs preserve the lattice orientation but alter the sublattice registry across the boundary. In the present case, the APBs are characterized by a lattice translation along (001) with a displacement vector $R=\frac{1}{2}\left[110\right]$ or $R=\frac{1}{2}\left[010\right]$ .

STEM image simulations (Dr. Probe^49^) superimposed on the experimental images show excellent agreement with both the pristine lattice (blue inset) and the APB-containing lattice (red inset). A second family of planar defects is observed in a different crystallographic orientation, most likely associated with the (110) plane family (Figure 2d). These defects are also identified as APBs, with displacement vectors $R=\frac{1}{2}\left[011\right]$ or $R=\frac{1}{2}\left[111\right]$ . The (001) APBs continue across intersections with the (110) APBs, undergoing a shift corresponding to the displacement vector of the intersecting boundary.

While the APBs observed here do not exhibit obvious wrong bonds in the idealized structure, local irregularities or terminations of the APBs within the grain, as highlighted in Figure 2d, may introduce dangling bonds. Additionally, subtle variations in local bonding geometry or coordination at the boundary cannot be excluded. Extended planar defects, including antiphase boundaries, are known to influence the electronic and optical properties of semiconductors and are therefore relevant for the optoelectronic behavior discussed in the following section.^45^ Additional TEM analysis of these defects is provided in Figures S5–S7.

STEM energy dispersive X-ray (EDX) analysis indicates a homogeneous and stoichiometric composition across the grain. No significant compositional variation is observed across antiphase boundaries (see Figure S8), indicating that large-scale compositional variation at these defects is absent within the resolution of the technique. However, more subtle atomic-scale variations cannot be excluded.

Strain analysis of the precession 4D-STEM data^50^ reveals no measurable strain (below ± 0.1%) across the Zn_3_P_2_ grain (see Figure S9), suggesting an effectively strain-relaxed lattice within the investigated region, consistent with growth on graphene via quasi–van der Waals epitaxy.

An atomic-resolution image of the Zn_3_P_2_/graphene/SiC interface (Figure 2f) reveals a sharp and well-defined interface in the investigated region. However, given the limited field of view, no conclusions can be drawn regarding the role of the substrate interface in the formation of APBs across the entire crystal. The formation mechanism of the high density of APBs remains an open question. Similar APBs have also been reported in Zn_3_P_2_ grown on InP substrates,^21^ indicating that they are not unique to quasi-van der Waals epitaxy on graphene. Furthermore, APBs are observed not only near the substrate interface but also within the overhanging regions of individual grains, suggesting that substrate morphology alone is unlikely to be the sole driving force for their formation. A detailed atomic-scale investigation of the electronic structure and formation of these APBs is beyond the scope of the present work and is currently being investigated using density functional theory calculations and advanced structural analysis.^51^

Overall, a dense network of antiphase boundaries with varying density and multiple orientations is observed within a single monocrystalline grain. While many APBs extend across the full thickness of the crystal, others exhibit local irregularities or terminations within the grain.

### Cathodoluminescence Emission Trends

To illustrate the optical properties of MOVPE-grown Zn_3_P_2_, the same grain analyzed by STEM in Figure 2 was investigated prior to lamella preparation using hyperspectral cathodoluminescence (CL) under varying excitation current and temperature conditions.

A representative SEM image of the mapped grain is shown in Figure 3a, together with spatial maps of the fitted emission peak area (b) and peak center energy (c), obtained from a 100 × 100 hyperspectral CL map acquired at 10 K (5 keV, 0.5 nA). The optical response is dominated by a single broad sub-bandgap emission with a pronounced low-energy tail, which was fitted using an exponentially modified Gaussian (EMG) function. The resulting maps therefore represent the spatial distribution of the peak center energy and integrated emission intensity.

**Figure 3:**
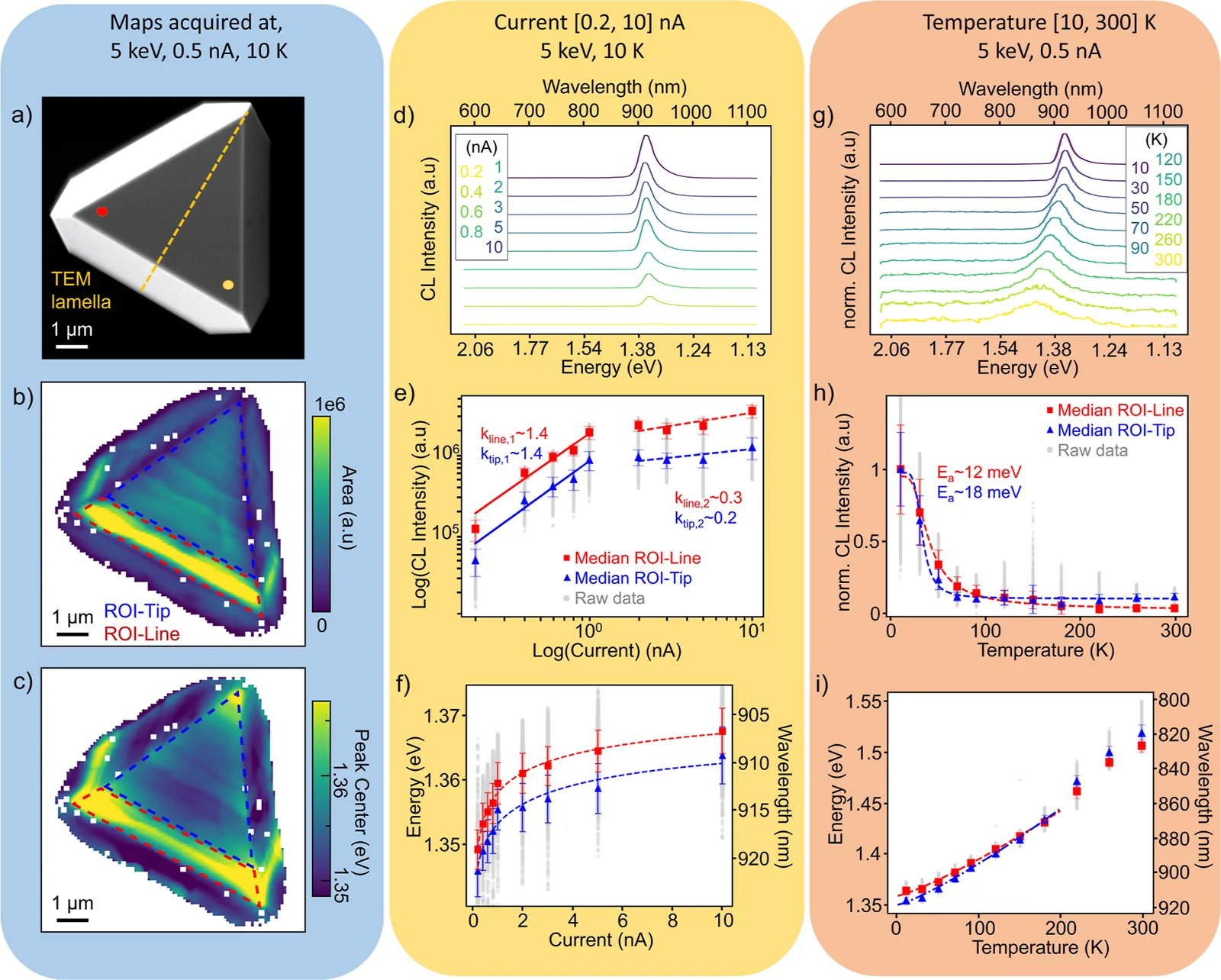
Cathodoluminescence (CL) analysis of MOVPE-grown Zn_3_P_2_, showing the spatial distribution and condition-dependent behavior of the broad near-infrared emission. Left panel (blue): Hyperspectral CL map (100 × 100 pixels) acquired at 10 K with a 5 keV electron beam and 0.5 nA current. (a) SEM image of the mapped grain, corresponding to the same grain analyzed by TEM in Figure 2a with indicated TEM lamella position. (b) Map of the fitted emission peak area. (c) Map of the fitted peak center energy. Two regions of interest (ROIs) are defined: a line-like region (ROI-Line, red dashed) and a tip region (ROI-Tip, blue dashed). Middle panel (yellow): Current-dependent CL measurements (0.2–10 nA) at 10 K. (d) Example spectra from a single pixel set at the yellow-marked spot in (a). (e) Median emission peak area as a function of beam current for both ROIs with power-law fits (*I* ∝ *P* ^*k*^). (f) Corresponding evolution of the fitted peak center energy. Right panel (orange): Temperature-dependent CL measurements (10–300 K) at 0.5 nA. (g) Example temperature series from a single pixel set at the red-marked spot in (a). (h) Median emission peak area versus temperature with Arrhenius fits. (i) Temperature dependence of the fitted peak center energy. Error bars represent the median absolute deviation within each ROI; gray points correspond to individual pixel values.

The ROI-Line region (red) exhibits higher emission intensity and an overall blueshift compared to the ROI-Tip region, coinciding with the highest APB density identified in the TEM analysis. In both regions, emission intensity and peak energy show spatial fluctuations across the APBs, while remaining comparatively uniform along directions parallel to them.

A more detailed correlation between APB density and the optical response is provided in Figure S10, where line-profile analysis and spatial overlays demonstrate that variations in both emission intensity and peak energy closely follow the APB distribution. While the correspondence is not strictly one-to-one, the overall trends indicate a strong, albeit not perfect, relationship between APBs and local optical variations.

As discussed in the structural analysis, the APBs correspond to a crystallographic lattice translation that preserves the overall crystal orientation while altering the sublattice registry. Despite the absence of a pronounced structural disruption, they are associated with clear variations in the local emission. In compound semiconductors, planar defects such as antiphase boundaries can induce significant electronic effects, particularly in polar materials where local phase transitions and associated polarization fields may lead to carrier localization and distinct luminescence features.^52–55^ Such mechanisms are not expected to apply in the present case, as Zn_3_P_2_ is nonpolar and no phase transformation is observed.

Instead, the observed optical variations are likely related to more subtle local perturbations associated with the APBs, such as variations in local bonding geometry, defect irregularities, or other modifications of the local atomic environment. Such perturbations could give rise to local potential fluctuations or modified recombination pathways, thereby influencing the emission intensity and peak energy. However, the present measurements do not distinguish between these possible microscopic mechanisms. As discussed above, a detailed atomic-scale investigation of the structure and electronic properties of these APBs is beyond the scope of the present work and is the subject of a dedicated complementary study.^51^

While the spatial variation in emission peak energy is most consistently explained by modulation of the local electronic potential associated with the APBs, variations in emission intensity are expected to be influenced by both the local electronic structure and the efficiency of radiative recombination. In addition to APB-associated perturbations, intensity variations may therefore arise from enhanced non-radiative recombination at APB terminations, local defect irregularities, or surface-related recombination. Atomic force microscopy and contrast-enhanced SEM images reveal regions with distinct surface textures, which may locally modify recombination rates and contribute to the observed intensity variations (see Figures S11 and S12). Low-temperature measurements further indicate that edges between {101} facets act as additional recombination sites due to their rough topography (Figure S13). The presence of such facets is consistent with a competition between kinetic and thermodynamic growth processes under nonequilibrium conditions, which can promote higher-energy facets alongside the stable {101} planes.^56^ As surface recombination is known to play a significant role in Zn_3_P_2_ but can be mitigated through surface passivation,^24,37,57^ implementing such strategies may provide a pathway to further improve the optical properties of Zn_3_P_2_ grown on graphene.

The general spectral evolution with excitation current at 10 K is shown in Figure 3d and with temperature in Figure 3g. At low temperature, the spectrum consists of a single broad emission peak with a pronounced low-energy tail. The spectral shape remains largely unchanged with increasing excitation current. With increasing temperature, however, the peak broadens and the high-energy side of the spectrum becomes less distinct. Above approximately 150 K, band-edge emission begins to emerge and partially overlaps with the defect-related peak, altering the apparent spectral shape. Such quenching of the band-edge emission below about 150 K is commonly reported for Zn_3_P_2_.^13,21,41^

Broad sub-bandgap emission is widely observed in Zn_3_P_2_ and is typically attributed to recombination involving acceptor states or band-tail–like transitions.^13,21,27,41,58^ Although several acceptor and deep defect states have been predicted for Zn_3_P_2_ in both experimental and theoretical studies, the spectra observed here are dominated by a single broad emission band, suggesting that the emission is governed by one dominant recombination channel. Considering the room-temperature bandgap of Zn_3_P_2_ (∼1.5 eV), the observed emission energies are consistent with recombination involving relatively shallow acceptor states or localized band-tail states located on the order of 100–200 meV above the valence band.

The excitation-current dependence of the CL intensity follows a power-law relation *I* ∝ *P* ^*k*^, where *I* is the emission intensity, *P* the excitation power, and *k* the exponent associated with the dominant recombination process.^59–62^ Both ROIs exhibit a similar behavior: at low excitation current, the intensity increases with *k* ≈ 1.4, followed by a strongly sublinear regime with *k* ≈ 0.2 at higher excitation current (Figure 3e). A slope around *k*–1.4 is often associated with defect-related transitions such as acceptor-to-band or band-tail recombination, while the sublinear regime at higher excitation reflects partial saturation of the involved states.^35,62–66^

Simultaneously, the emission peak exhibits a progressive blueshift with increasing excitation current in both ROIs (Figure 3f), eventually approaching saturation at higher excitation densities. Such behavior is widely reported in defect-rich semiconductors and is typically attributed to the filling of localized states or the screening of potential fluctuations.^35,63–65^

The temperature dependence of the emission intensity was analyzed using an Arrhenius model (Figure 3h). The extracted activation energies are 12 meV for the red ROI-Line region and 18 meV for the blue ROI-Tip region. Although the two regions show slightly different activation energies, the difference is small and does not indicate distinct recombination channels. Instead, the activation energy likely reflects the effective barrier for thermally activated escape from the radiative recombination pathway rather than the binding energy of the radiative state itself. In systems dominated by localized or band-tail states, carriers may thermally redistribute between nearby localized states or escape toward non-radiative recombination centers, resulting in relatively small apparent activation energies.^45^ The extracted values (≈10–20 meV) are therefore significantly smaller than the binding energies of shallow acceptors predicted for Zn_3_P_2_, which are typically reported to lie above 100 meV above the valence band.^13,24,25,67^

The peak center energy increases with temperature for both ROIs (Figure 3i). Such blueshifting behavior has been widely reported for recombination involving localized or band-tail states, where carriers thermally escape from deeper localized states and recombine from progressively shallower states as temperature increases.^35,63–66^

Overall, the similar excitation- and temperature-dependent behavior, together with identical line widths across both regions (Figure S14) and the temperature-dependent homogenization of the emission intensity (Figure S15), indicate that the same dominant recombination mechanism governs the optical response throughout the grain. The spatial variation in emission energy is most consistent with local modulation of the electronic potential landscape, whereas the emission intensity may additionally be influenced by variations in local recombination efficiency. The present measurements establish a clear correlation between APB density and the local optical response, but they do not directly identify the microscopic mechanism responsible for this behavior.

While the exact nature of the radiative recombination pathway cannot be unambiguously identified, the collective experimental observations are most consistent with an interpretation involving localized band-tail states. In particular, the continuous spatial variation of the emission energy, the persistence of a single broad asymmetric emission band across all investigated conditions, the absence of distinct line width differences, and the characteristic excitation- and temperature-dependent blueshifts are all consistent with recombination occurring within a spatially varying potential landscape. First-principles and density functional theory calculations indicate that zinc vacancies have among the lowest formation energies in Zn_3_P_2_,^24,25^ making zinc vacancies plausible candidates for the shallow acceptor state in the proposed tail-to-acceptor recombination pathway. Nevertheless, alternative radiative recombination pathways, including donor–acceptor pair (DAP) recombination, cannot be fully excluded based on the present steady-state measurements. Complementary spectrally resolved time-resolved optical measurements could provide additional insight into recombination dynamics and help further discriminate between the proposed recombination pathways.

Overall, the CL spectra exhibit a single broad emission band, indicating that the optical response is dominated by a common recombination mechanism across the investigated regions. The recombination is most consistent with a single dominant pathway involving transitions between conduction-band tail states and shallow acceptors, as illustrated schematically in Figure 4. This assignment is supported by the excitation- and temperature-dependent behavior: increasing excitation density leads to progressive filling of localized states and a shift of the emission toward higher energies, while increasing temperature promotes thermal redistribution of carriers toward shallower states, resulting in the observed blueshift of the emission peak. Such behavior is commonly associated with recombination involving localized band-tail states. Although additional pathways, such as band-to-band recombination (observed under PL conditions) and defect-assisted non-radiative recombination, may be present, they do not dominate under the CL conditions investigated here.

**Figure 4:**
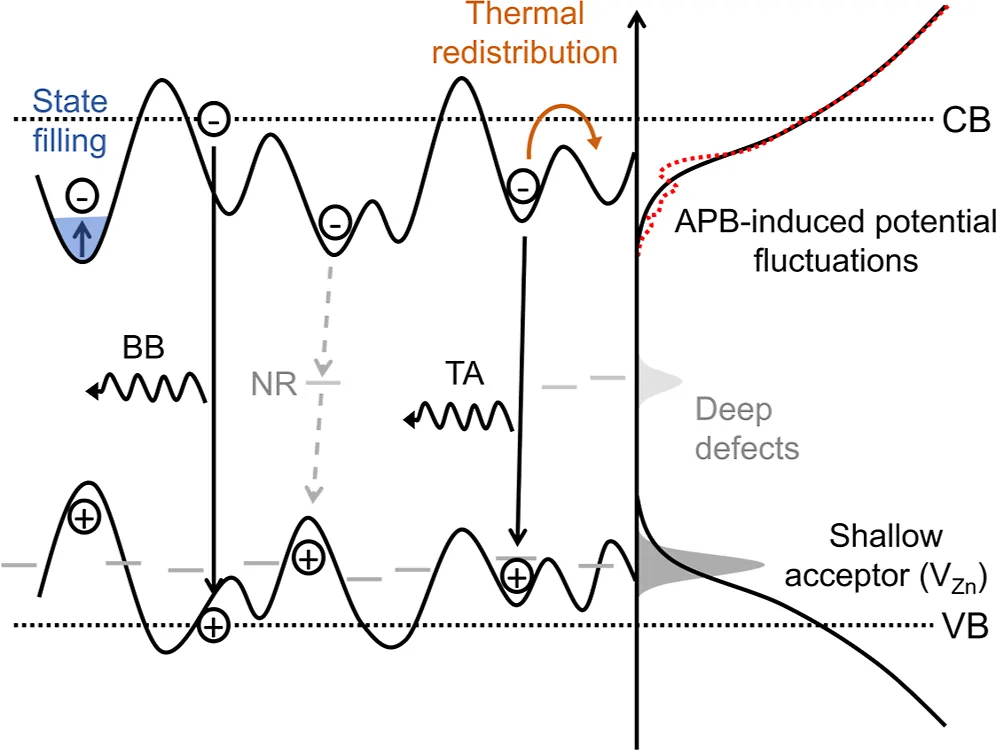
Schematic illustration of the recombination pathway most consistent with the present cathodoluminescence observations. The main recombination pathways are shown, including band-to-band (BB) and tail-to-acceptor (TA) radiative transitions, as well as non-radiative recombination (NR) via deep defect states. Increasing excitation density leads to state filling and recombination from higher-energy localized states, while increasing temperature promotes thermal redistribution of carriers toward shallower states, resulting in a blueshift of the emission peak. One possible interpretation is that APB-associated perturbations locally modify the electronic potential landscape.

### Grain Boundary Recombination and Carrier Diffusion

Having established the recombination behavior within individual grains, the role of grain boundaries (GBs) as potential non-radiative recombination centers were investigated using spatially resolved cathodoluminescence.

Figure 5a shows two merged Zn_3_P_2_ grains grown at 375 °C and V/II = 41.7, analyzed by hyperspectral CL mapping between 10 and 300 K. The corresponding CL intensity maps at 10 and 300 K are shown in Figure 5b,c. As observed previously for individual grains, line-like intensity variations are visible within the grains. However, at all temperatures, a pronounced decrease in emission intensity occurs at the grain boundary (GB), marked by the dashed red line.

**Figure 5:**
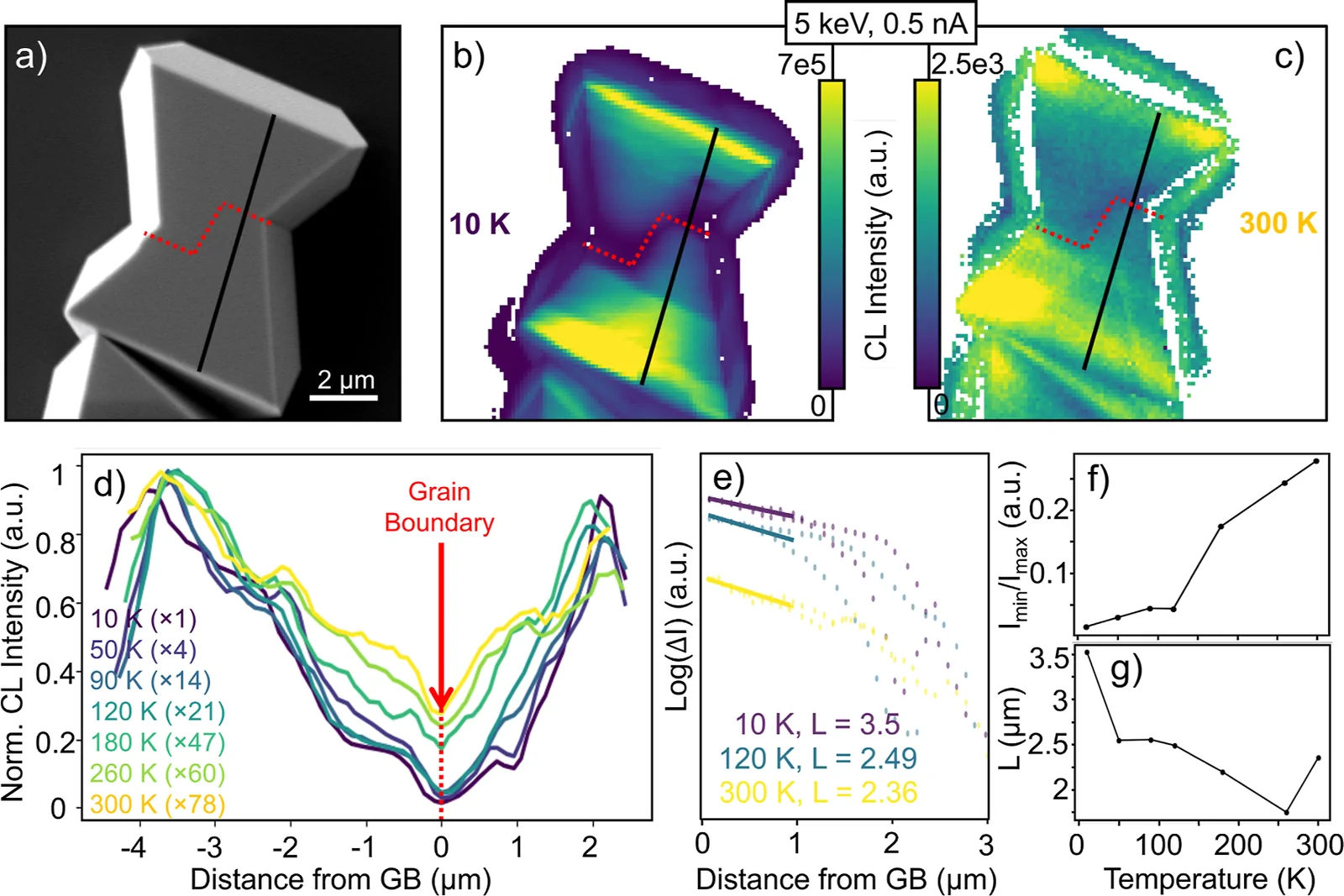
Cathodoluminescence analysis of a grain boundary (GB) between two merged Zn_3_P_2_ grains grown by MOVPE. Hyperspectral CL maps were acquired at 5 keV and 0.5 nA. (a) SEM image of the mapped region with the line scan indicated. (b,c) Integrated CL intensity maps at 10 and 300 K, respectively, showing strong emission quenching at the grain boundary. (d) Normalized CL intensity profiles across the GB for different temperatures. (e) Recovery of the CL intensity away from the GB plotted as Δ*I* = *I*_∞_ − *I*(*x*) for selected temperatures, used to extract the effective diffusion length. (f) Ratio of minimum to maximum CL intensity across the GB as a function of temperature. (g) Extracted recovery length *L* as a function of temperature.

The intensity evolution along the line indicated in Figure 5a is shown in Figure 5d for all temperatures. A clear minimum in CL intensity is observed at the GB position, indicating strong non-radiative recombination at the boundary. Although the contrast decreases with increasing temperature (Figure 5f), the CL intensity at the GB remains reduced by at least *a* factor of 3 across all temperatures. Similar emission quenching at grain boundaries has been reported in other thin-film photovoltaic materials such as CIGS, CdTe, kesterites and perovskites, where GB recombination can significantly impact device performance.^30–36^ The reduction in GB contrast with increasing temperature indicates a temperature-dependent modification of the recombination landscape, suggesting the activation of additional recombination pathways that compete with the GB recombination channel.

The spatial recovery of the CL intensity away from the grain boundary is analyzed using a steady-state diffusion model. For a strong non-radiative sink at the GB, the carrier density and therefore the CL intensity recover exponentially with distance according to

$$I\left(x\right)=I_{\infty }\left(1-e^{-x/L}\right)$$

where *I*_∞_ is the intensity far from the GB and *L* is an effective recovery length characterizing the spatial recovery of the cathodoluminescence signal. In the strong sink limit, this characteristic length scale follows the same functional dependence as the diffusion length, $L_{diff}=\sqrt{D\tau_{eff}}$ , as obtained from the steady-state solution of the diffusion equation with recombination,^68^ where *D* is the diffusion coefficient and τ_eff_ the effective carrier lifetime.

The recovery behavior is analyzed by plotting the intensity deficit Δ*I* = *I*_∞_ − *I*(*x*) as a function of distance from the GB (Figure 5e). Excluding finite-size effects near the grain edges, the intensity deficit follows an approximately exponential recovery. Fitting the near-GB region yields an effective recovery length of approximately 2–3 μm between 10 and 300 K (Figure 5g). The extracted effective recovery length is comparable in magnitude to the minority carrier diffusion lengths reported for Zn_3_P_2_ (5–7 μm).^14,15^ However, both the reported diffusion lengths and transport properties of Zn_3_P_2_ span a wide range depending on the measurement technique, injection regime, crystal quality, and growth method. For example, while relatively low mobilities have been reported for bulk and polycrystalline material,^23,69^ recent selective-area epitaxial Zn_3_P_2_ has demonstrated hole mobilities exceeding 560 cm^2^V^−1^ s^−1^.^70^ Consequently, the present measurements, which probe ambipolar transport under high-injection conditions, can only provide a qualitative comparison of transport length scales and should not be interpreted as a direct quantitative measure of the minority carrier diffusion length.

In the present analysis, the grain boundary is treated as an ideal non-radiative sink, such that the recovery profile is described by a single characteristic length scale *L*. While more elaborate models can, in principle, extract the grain-boundary recombination velocity by analyzing both the recovery slope and amplitude,^71^ this requires independent knowledge of the bulk carrier lifetime and transport parameters, which are not available for the present samples. The analysis is therefore restricted to the effective recovery length *L*. Future time-resolved cathodoluminescence measurements could provide direct access to the carrier lifetime and thereby enable a more quantitative determination of the grain-boundary recombination velocity.

Interestingly, the extracted recovery length remains approximately constant or slightly decreases with increasing temperature (Figure 5g). This behavior can be rationalized within the relation for *L*. In semiconductors, the effective carrier lifetime τ_eff_ typically decreases with increasing temperature due to enhanced phonon-assisted and defect-mediated non-radiative recombination.^68^ To maintain an approximately constant *L*, this reduction in τ_eff_ must be compensated by an increase in the diffusion coefficient *D*. Such an increase can arise from enhanced carrier transport at elevated temperatures, for instance due to thermally activated delocalization of carriers from localized or band-tail states, consistent with the temperature-dependent transport behavior reported for polycrystalline Zn_3_P_2_ thin films.^41^ This interplay provides a consistent explanation for the reduced GB contrast observed at elevated temperatures (Figure 5f), as increased carrier mobility promotes a more spatially extended redistribution of carriers, while the reduced lifetime enhances non-radiative recombination, in agreement with the activation of additional recombination pathways discussed above.

Grain boundaries (GBs) in Zn_3_P_2_ have previously been identified as charged, recombination-active regions that can dominate carrier transport.^37^ In agreement with this picture, the pronounced CL quenching observed at GBs demonstrates that they act as efficient non-radiative recombination centers. The extracted effective recovery length, which approaches a significant fraction of the grain size, suggests that carrier diffusion toward these non-radiative recombination regions leads to a spatial reduction in carrier density near the GBs. The presence of grain-boundary charge and associated electrostatic barriers further modifies the local carrier distribution. Since the quasi-Fermi level splitting is directly governed by the local electron and hole populations, this results in a corresponding spatial variation and reduction of the quasi-Fermi level splitting in the vicinity of GBs.^68,72^

Although some earlier studies reported a limited impact of GBs on device performance,^16^ these observations can be reconciled with the present results by considering differences in microstructure. In particular, those studies were conducted on materials with substantially larger grain sizes, where the lower grain-boundary density likely mitigated their overall impact. In the present case, the pronounced CL quenching together with an effective recovery length comparable to the grain size suggests that grain boundaries can influence carrier transport and recombination over a substantial fraction of the absorber volume. Similar recovery lengths have been observed from room-temperature photoluminescence measurements, as shown in Figure S16.

These observations therefore suggest that reducing grain-boundary density and activity, for example through larger grain growth or grain-boundary passivation, is likely to be an important pathway toward improving the optoelectronic quality of Zn_3_P_2_ absorbers.

### MBE vs MOVPE Thin-Film Quality

The analyses above reveal how defects within grains and grain boundaries shape the local optoelectronic behavior of Zn_3_P_2_. In the following, these insights are used to compare the properties of thin films grown by MBE and MOVPE, highlighting how differences in growth conditions influence defect-related sub-bandgap emission and grain structure.

Figure 6 compares the CL emission at 10 K of coalesced Zn_3_P_2_ thin films grown by MBE and MOVPE under markedly different conditions. The MBE sample was grown at a nominal temperature of 230 °C with a V/II ratio of 3.4, while the MOVPE sample was grown at 375 °C with a V/II ratio of 41.7. In both cases, the film thickness exceeds the electron interaction volume under the CL conditions, ensuring that the measured emission is representative of the bulk film. The samples are therefore representative of the respective growth approaches: MBE films show limited variation across the explored growth window, while the MOVPE sample corresponds to the only fully coalesced thin film obtained under the investigated conditions. It should be noted that the V/II values are not directly comparable due to differences in precursor chemistry and growth dynamics between the two techniques.

**Figure 6:**
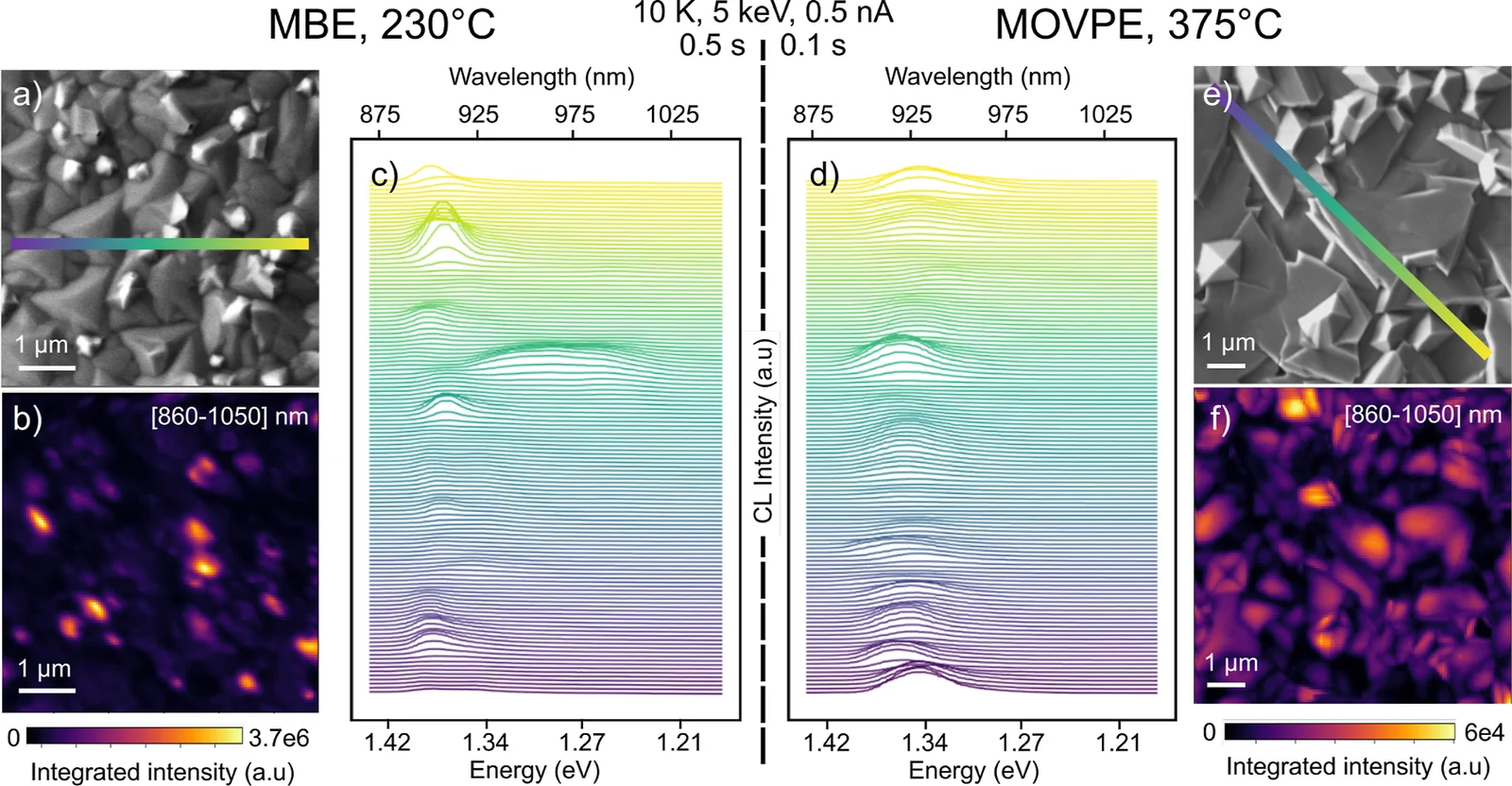
Comparison of cathodoluminescence of Zn_3_P_2_ thin films grown by MBE (left) and MOVPE (right) at 10 K. (a) SEM image of the MBE sample. (b) Integrated CL intensity map in the spectral range 860–1050 nm of the CL map acquired at 10 K for the same region as shown in the SEM image in (a). (c) CL spectra extracted along the line indicated in (a), plotted with an offset for clarity. The color coding corresponds to the spatial position along the scan direction. (d) CL spectra along the line indicated in (e) for the MOVPE sample, displayed with the same offset and color scheme as in (c) for direct comparison. (e) SEM image of the second sample with the corresponding line scan indicated. (f) Integrated CL intensity map (860–1050 nm) of the region shown in the SEM image in (e). All CL measurements were performed at 10 K with a 5 keV beam energy and 0.5 nA current. MBE sample pixels were acquired for 0.5 s and MOVPE for 0.1 s.

Measurements on the MBE-grown film were performed on an exfoliated flake detached from the graphene substrate, similar to previously reported measurements.^27^ The MOVPE-grown film, in contrast, was measured while still in contact with the graphene layer. Together with possible differences in film thickness, these factors influence the detected CL intensity, as contact with conductive substrates such as graphene can lead to partial quenching of the emission intensity due to carrier extraction or interface recombination.^27^ Consequently, the absolute CL intensities between the two samples cannot be directly compared, and the following discussion focuses primarily on spatial emission variations and spectral features.

Two main emission bands can be identified in the MBE-grown film. The first, centered around 1.37 eV, is referred to as the high-energy (HE) emission, while a second band appears below approximately 1.34 eV and is denoted as the low-energy (LE) emission. The waterfall plot of spectra extracted along the line shown in Figure 6a (Figure 6c) reveals strong spatial variations in both peak position and relative intensity for the HE and LE emissions, indicating significant local variations in the optical properties of the film, with the LE emission being highly localized. Similar spatially localized low-energy emission has been reported previously in MBE-grown Zn_3_P_2_ thin films on graphene.^27^

In contrast, the MOVPE-grown thin film exhibits only the emission previously discussed in Figure 3, centered around 1.35 eV. As shown in the spectral evolution along the line scan in Figure 6d, spatial variations in intensity and peak position are still observed, likely related to APBs and grain boundaries, but no additional low-energy emission band is present.

Despite differences in peak position and line width between the HE emissions of the MBE- and MOVPE-grown films, their similar temperature- and current-behavior suggests that they originate from the same underlying recombination mechanism (see Figures S17 and S18 for temperature- and current-behavior of MBE-grown thin films). The observed spectral shift and broadening are likely related to differences in band-tail states, composition, or strain arising from the distinct growth conditions.

The strong spatial localization of the LE emission suggests that it originates from local variations in the electronic structure. Previous studies on comparable Zn_3_P_2_ thin films have reported the presence of highly localized strain fields,^27^ which could give rise to such emission features. Density functional theory calculations (Figure S19) further predict an anisotropic reduction of the Zn_3_P_2_ band gap under strain, with the magnitude of the shift depending strongly on the crystallographic direction. The predicted energy shifts are of the same order as the experimentally observed separation between the high- and low-energy emission bands, indicating that local strain is a likley origin of the additional low-energy emission.

Comparison of the SEM image and the integrated CL map for the MOVPE-grown film (Figure 6e,f) reveals a clear correlation between morphology and emission intensity, with reduced CL intensity occurring predominantly at grain boundaries and grain edges. This behavior contrasts with the MBE-grown film, where strong emission variations occur within the grains.

Overall, the absence of the strain-related low-energy emission in the MOVPE-grown thin film indicates a reduced density of localized strain fields and associated defect states compared to the MBE-grown material. Together with the clear correlation between morphology and emission intensity, this suggests that MOVPE growth produces material with improved optoelectronic quality and fewer non-radiative defects within the grains. This improvement is attributed in part to the higher growth temperatures accessible in MOVPE, which enhance adatom mobility and promote defect relaxation during growth. In contrast, MBE growth of Zn_3_P_2_ is inherently limited to significantly lower substrate temperatures due to the high desorption rates of both Zn and P_2_,^73^ which restricts surface diffusion and favors the incorporation of local strain and structural defects. As a result, the extended adatom diffusion lengths achievable in MOVPE likely play a key role in reducing the formation of localized strain fields and associated band-tail states at grain boundaries.

## Conclusion

In this work, we demonstrate the growth of Zn_3_P_2_ on graphene by metal–organic vapor phase epitaxy (MOVPE) and establish a spatial correlation between structural defects and local optical response through correlative transmission electron microscopy and cathodoluminescence spectroscopy. In situ treatment of the graphene substrate with HCl_(g)_ prior to growth improves the crystal quality of the resulting films, highlighting the importance of surface preparation for achieving high-quality Zn_3_P_2_ layers. Further investigation of the MOVPE growth window may enable improved control of nucleation and the development of more homogeneous films over larger areas.

Transmission electron microscopy reveals that individual grains are monocrystalline and contain a high density of antiphase boundaries. Within the grains, no significant elemental composition or strain is observed, including around the APBs. This behavior is consistent with quasi–van der Waals epitaxy, where weak interfacial coupling relaxes lattice-matching constraints.

Correlative CL measurements performed on the same grain reveal spatial variations in emission intensity and peak energy that coincide with the density of APBs. These observations indicate that APB-rich regions are associated with local variations in the optical response, consistent with modulation of the local potential landscape, although the microscopic origin of this correlation remains to be established. Temperature- and current-dependent measurements indicate that the dominant sub-bandgap emission is most consistent with recombination involving band-tail states and shallow acceptors.

Spatially resolved CL analysis of grain boundaries reveals pronounced changes in non-radiative recombination, with an effective recovery length of approximately 3 μm. This recovery length is comparable to roughly half the grain size, suggesting that grain boundaries can influence carrier transport and recombination dynamics over large areas in Zn_3_P_2_.

Finally, extending this analysis to the thin-film level, comparison between MBE- and MOVPE-grown samples reveals clear differences in their optical properties. While MBE-grown films exhibit additional spatially localized low-energy emission attributed to strain-related band-tail states, MOVPE-grown films show only a single emission peak present across the entire film, together with a clear correlation between morphology and emission intensity. The absence of strain-related sub-bandgap emission suggests that MOVPE growth reduces the formation of localized strain fields and associated defect states at grain boundaries.

Overall, MOVPE-grown films exhibit larger grains and the suppression of the additional localized low-energy emission observed in MBE-grown material, indicating improved optoelectronic material quality compared to MBE growth. At the same time, the clear identification of grain boundaries as dominant non-radiative recombination centers highlights the importance of controlling the microstructure of polycrystalline Zn_3_P_2_.

These results highlight that improving grain size and mitigating grain-boundary recombination, for example through optimized growth conditions or targeted passivation strategies, represents a promising pathway toward high-performance earth-abundant Zn_3_P_2_ photovoltaic absorbers.

## Experimental Methods

### Zn_3_P_2_ Growth Procedure

Zn_3_P_2_ was grown by molecular beam epitaxy (MBE) and metal–organic vapor phase epitaxy (MOVPE).

### MBE Growth

MBE growth was performed in a Veeco GENxplor system. Zn (6N purity) was supplied from a valved cracker cell, while P_2_ was provided by a valved GaP (6N purity) source (MBE-Komponenten) equipped with an integrated Ga trapping system. The V/II flux ratio (P_2_/Zn) was determined from beam flux monitor measurements performed immediately after growth.

The substrate temperature was measured using a thermocouple positioned above the manipulator; all reported temperatures are nominal values. Prior to growth, the substrates were annealed under ultrahigh vacuum (UHV, below 5.0 × 10^−10^ Torr) at 650 °C for 2 h in a preparation chamber adjacent to the growth module to desorb water and airborne contaminants. An additional 1 h annealing step at 650 °C was performed in the growth chamber.

After annealing, the substrate was cooled to the growth temperature and exposed to P_2_ for 5 min before initiating Zn_3_P_2_ growth. The beam equivalent pressures (BEP) were 1.4 × 10^−6^ Torr for Zn and 3.4 × 10^−7^ Torr for P_2_. Growth duration was 390 min.

### MOVPE Growth

MOVPE growth was carried out in an Aixtron 3 × 2″ close-coupled showerhead (CCS) MOCVD reactor operating at 100 mbar with a total flow rate of 8000 sccm hydrogen carrier gas.

Prior to growth, the graphene substrates were degassed at 650 °C for 15 min under a PH_3_ partial pressure of 0.1 mbar, with or without additional HCl_(g)_ flow of 1.76 × 10^−3^ mbar. The reactor was then cooled to the growth temperature (350 or 375 °C). Once thermal stabilization was reached, Zn_3_P_2_ growth was initiated using diethylzinc (DEZn) as the Zn precursor and PH_3_ as the P precursor. The DEZn partial pressure was 7.19 × 10^−3^ mbar, while the PH_3_ partial pressure ranged from 2.25 × 10^−1^ mbar to 3.0 × 10^−1^ mbar. Growth durations ranged from 120 to 210 min.

### Graphene Substrates

Zn_3_P_2_ was deposited on epitaxial monocrystalline graphene grown on the Si-face of 6H–SiC by chemical vapor deposition (CVD) under hydrogen.^74^ The graphene was synthesized at 1550 °C under low hydrogen partial pressure,^75^ under low hydrogen partial pressure, resulting in the self-limited growth of monocrystalline monolayer graphene with approximately 5% bilayer coverage.

A carbon buffer layer composed of sp^2^ and sp^3^ hybridized carbon is present at the graphene/SiC interface. The SiC substrates had an average miscut of approximately 0.07°, leading to step heights between 0.25 and 1.5 nm and terrace widths up to 400 nm.

### Measurement Techniques

#### Scanning Electron Microscopy (SEM)

SEM images were acquired using a Zeiss Merlin field-emission SEM equipped with a Gemini column or a Zeiss Gemini 500. Imaging was performed using an in-lens secondary electron detector at an acceleration voltage of 2 or 3 keV and a beam current of 80 pA.

#### Atomic Force Microscopy (AFM)

AFM measurements were carried out using a Bruker FastScan system operated in tapping mode. Bruker FluidScan + probes were used for all measurements.

#### Photoluminescence Spectroscopy (PL)

Room-temperature PL measurements were performed using a Renishaw inVia system with a 532 nm excitation laser and a 100× objective (NA = 0.85), corresponding to an illumination intensity of approximately 393 μW/μm^2^. The laser power was 180 μW for all measurements. Single-point spectra were acquired with 1 s integration time and averaged over 60 accumulations, whereas mapping measurements were performed with a single acquisition per pixel. A 300 lines mm^−1^ grating was used.

#### Raman Spectroscopy

Raman measurements were performed at room temperature using the same Renishaw inVia setup with a 532 nm excitation laser and a 100× objective (NA = 0.85). Spectra were acquired with 5 s integration time and averaged over 60 accumulations. The laser power was approximately 180 μW. An 1800 lines mm^−1^ grating was used.

#### Cathodoluminescence Spectroscopy (CL)

Temperature-dependent CL measurements (10–300 K) were conducted using an Attolight Allalin system. Spectra were recorded with an Andor Newton DU920P-BEX2-DD Si-CCD detector at an acceleration voltage of 5 keV, beam currents between 0.2 and 10 nA, and acquisition times ranging from 0.1 to 0.5 s per pixel. A 150 lines mm^−1^ grating with a blaze wavelength of 500 nm was used.

CL spectra were corrected by subtracting the CCD dark count. To reduce high-frequency noise, a Savitzky–Golay low-pass filter (window size = 5 pixels, polynomial order = 3, derivative = 0) was applied.

When fitting was performed, emission peaks were modeled using an exponentially modified Gaussian (EMG) function, which reproduces the asymmetric peak shape observed in the spectra. The extracted fit parameters were analyzed using median values rather than arithmetic means to obtain robust representative values. The median was selected because it is less sensitive to skewed distributions and rare high-intensity events that can bias the mean. For each defined region of interest (ROI), the upper 5% of the parameter distribution was excluded prior to calculating the median. This approach reduces the impact of localized bright emission spots or occasional fitting artifacts while preserving the data set’s statistically relevant behavior.

#### Transmission Electron Microscopy (TEM)

TEM samples were prepared using a lift-out technique on a Zeiss CrossBeam 550 dual-beam focused ion beam-SEM (FIB-SEM) instrument. TEM experiments were conducted on a double-corrected FEI Titan Themis microscope at 300 kV. Atomic-resolution scanning transmission microscopy (STEM) images used a convergence angle of 20 mrad, a beam current of 100 pA, and collection angles of >50 mrad and <20 mrad for HAADF and BF images, respectively. For EDX, the beam current was increased to 270 pA, and the characteristic X-rays were collected with silicon drift detectors (super-X system). For the precession 4D-STEM experiment, a NanoMEGAS Topspin system was used, with data collected using a Quantum Detectors MerlinEM direct-detection camera at a convergence angle of 0.75 mrad, a beam current of 470 pA, a camera length of 230 mm, and a precession angle of 1°. The entire Zn_3_P_2_ grain was mapped by stitching two 4D-STEM maps with a 15 nm pixel size and a 6 ms dwell time. Strain maps were processed using in-house codes developed as a plugin for GMS software, named 4Dview, which appears to be quite robust against artifacts from diffraction contrasts. Multislice atomic-resolution STEM image simulations were performed with Dr Probe software,^49^ using a 105 nm-thick crystal sample from the CIF file of P4_2_/nmc Zn_3_P_2_, which has been modified to introduce the specific antiphase boundary defect.

#### Density Functional Theory (DFT)

Ab initio calculations were performed using the Vienna Ab initio Simulation Package (VASP, version 6.3.2).^76,77^ The initial crystal structure of Zn_3_P_2_ was obtained from the Materials Project (mp-2071).^78^ Strain was applied via linear deformations in increments of 0.5% over a range from −3% to +3% along three directions orthogonal to the [1̅11] plane. Ionic positions were fully relaxed using the PBEsol exchange–correlation functional,^79^ with a plane-wave cutoff energy of 350 eV, until the residual forces on all atoms were below 1 meV/Å. Brillouin zone integrations were performed using a Γ-centered 9 × 9 × 6 *k*-point mesh. Subsequently, electronic band structures were calculated along high-symmetry paths in reciprocal space using 278 *k*-points and the modified Becke–Johnson potential.^80^ The same cutoff energy of 350 eV was employed, and both spin–orbit coupling and nonspherical contributions to the charge density were included.

## Supplementary Material



## Data Availability

The data supporting the findings of this study are available at 10.5281/zenodo.20178154.

## Notes

Supporting Information includes additional growth optimization, graphene characterization, transmission electron microscopy, strain and EDX analysis, cathodoluminescence and photoluminescence measurements, surface morphology analysis, density functional theory calculations, and supplementary figures (PDF)
