# Supplementary material for: Cathodoluminescence Analysis of Defects and Grain Boundaries in Zn3P2 Thin Films Grown on Graphene by MOVPE and MBE
Source: ACS Mater Au. 2026 Jul 28;6(5):1172–85. doi: 10.1021/acsmaterialsau.6c00120 (PMC13580687; doi:10.1021/acsmaterialsau.6c00120)
Supplement: Supplementary file 1 [file mg6c00120_si_001.pdf]

Supplementary Information for : Cathodoluminescence Analysis of Defects and Grain Boundaries in Zn_3P_2 Thin Films Grown on Graphene by MOVPE and MBE

Thomas Hagger[1], Mohammadreza Hassanzadeh[1], Aidas Urbonavicius[2], Ahmed El Alouani[3], Victor Boureau[4], Gulnaz Ganeeva[4], Nico Kawashima[5,6], Raphael Lemerle[1], Kamil Artur Wodzislawski[1], Sebastian Lehmann[2], Kimberly A. Dick[2], Silvana Botti[5,6], Adrien Michon[3], Anna Fontcuberta i Morral[1], and Simon Escobar Steinvall[2]

[1]Laboratory of Semiconductor Materials, Institute of Materials, School of Engineering, École Polytechnique Fédérale de Lausanne (EPFL), 1015 Lausanne, Switzerland
[2]Centre for Analysis and Synthesis and NanoLund, Lund University, Box 124, 221 00 Lund, Sweden
[3]Université Côte d'Azur, CNRS, CRHEA, Rue Bernard Grégory, 06560 Valbonne, France
[4]Interdisciplinary Center for Electron Microscopy (CIME), EPFL, 1015 Lausanne, Switzerland
[5]Research Center Future Energy Materials and Systems of the University Alliance Ruhr and ICAMS, Ruhr University Bochum, Universitätsstraße 150, D-44801 Bochum, Germany
[6]Institut für Festkörpertheorie und-optik, Friedrich-Schiller-Universität Jena, Max-Wien-Platz 1, 07743 Jena, Germany

Corresponding authors: anna.fontcuberta-morral@epfl.ch; simon.escobar_steinvall@chem.lu.se

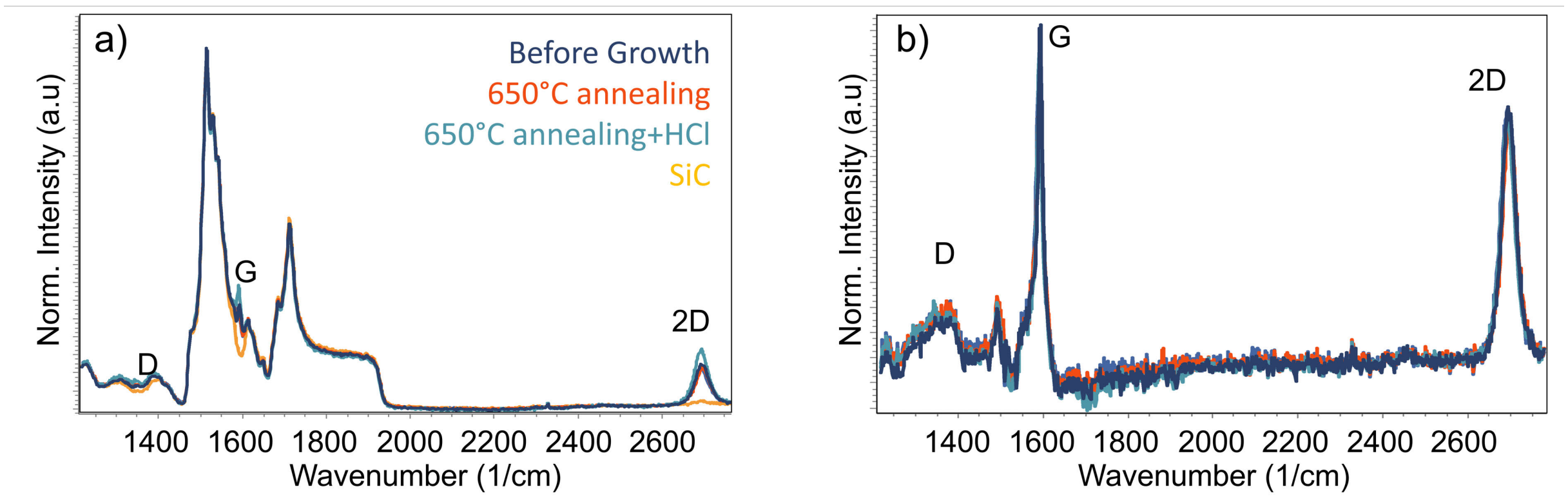

Figure S1: Raman spectra of the graphene substrate before and after the growth of Zn_3P_2 by MOVPE. (a) shows the raw spectra and (b) the graphene spectra with subtracted SiC background. No damage to the graphene was induced during any of the processes, as evidenced by the unchanged intensity of the D peak.

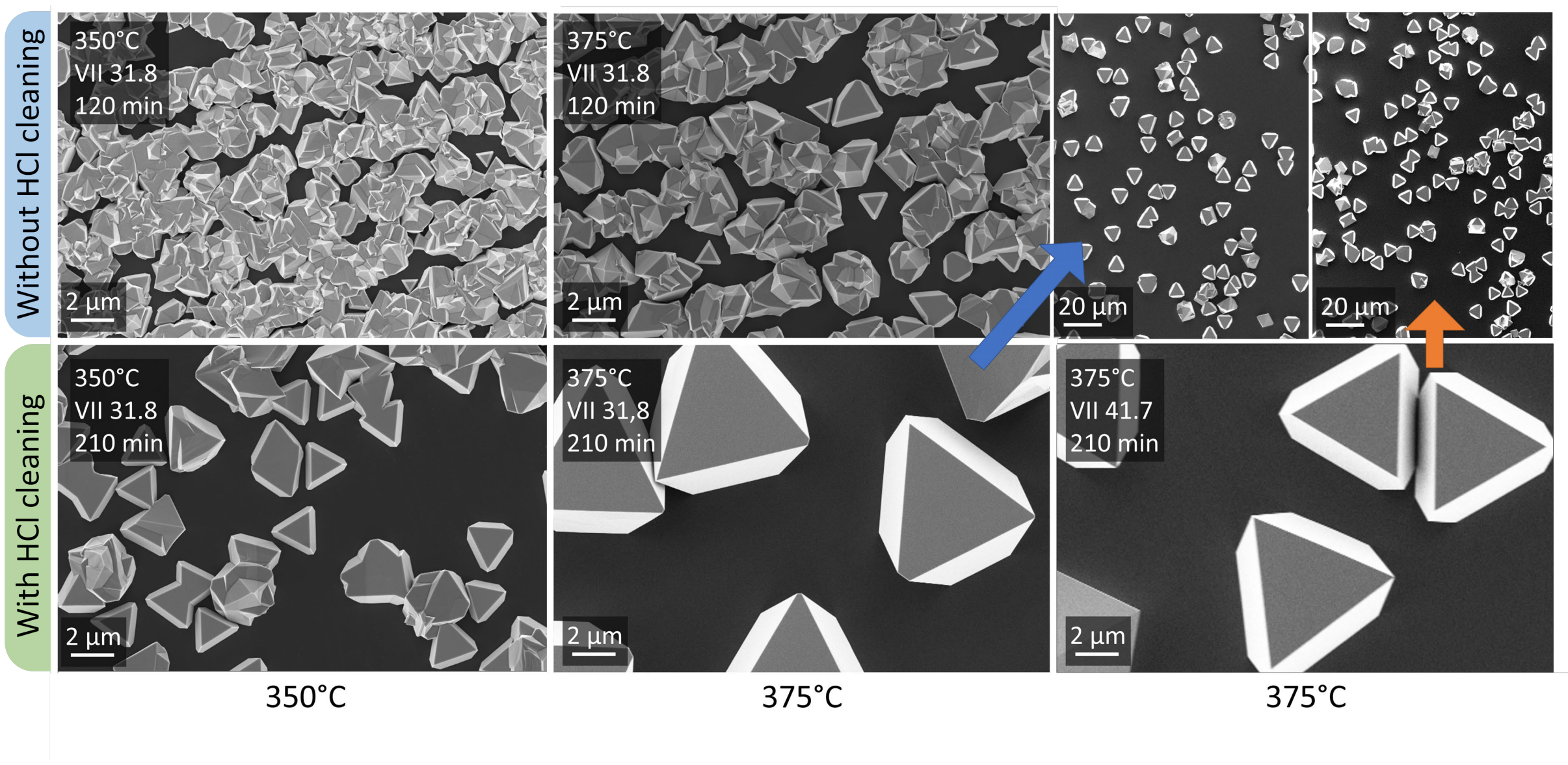

Figure S2: MOVPE growth outcome of Zn_3P_2 as presented in the main text with an additional increase in V/II flux ratio. No significant changes in nucleation density are observed by increasing the V/II flux ratio.

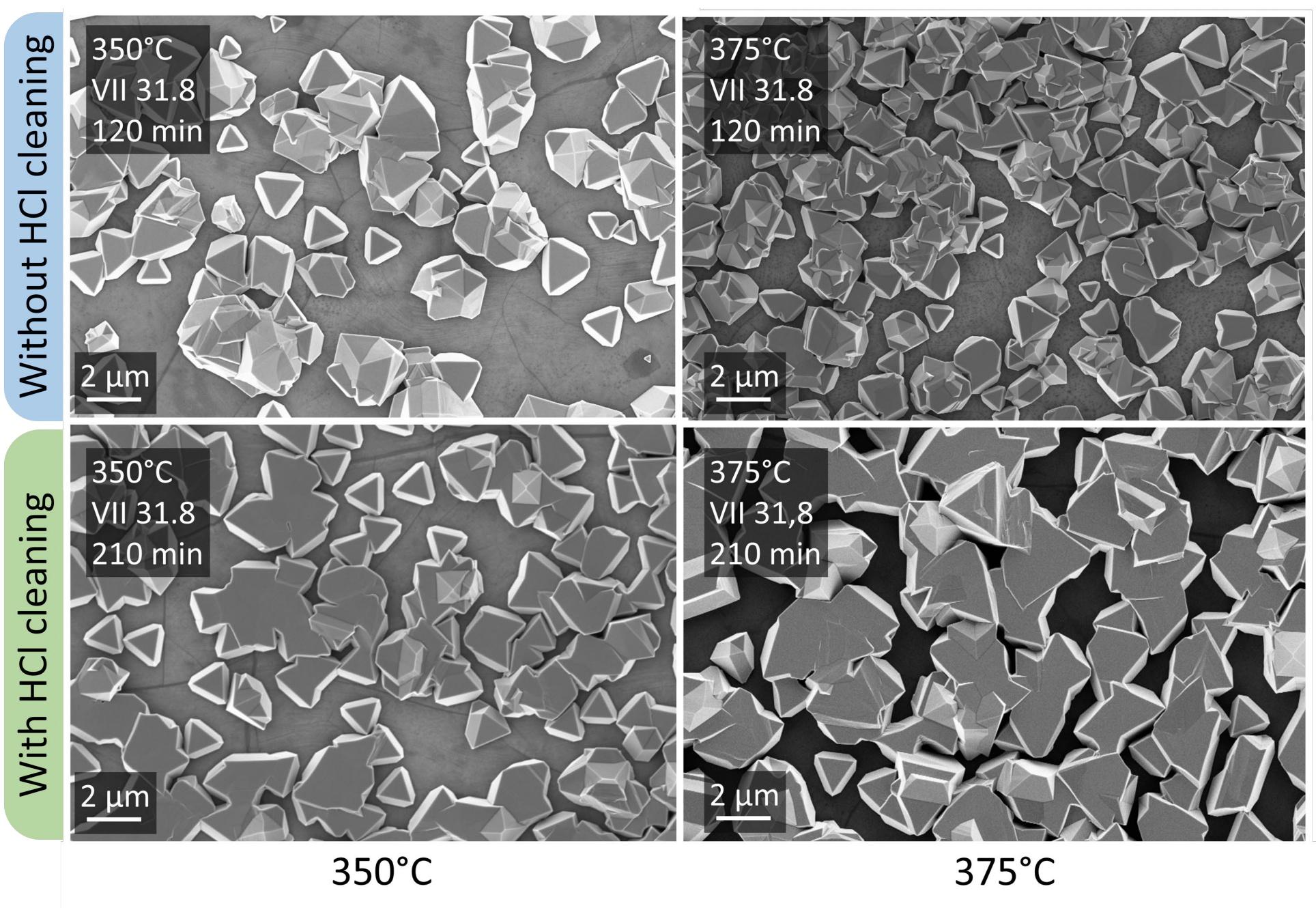

Figure S3: MOVPE growth outcome of Zn_3P_2 on the commercially available wet-transferred graphene from Graphenea.

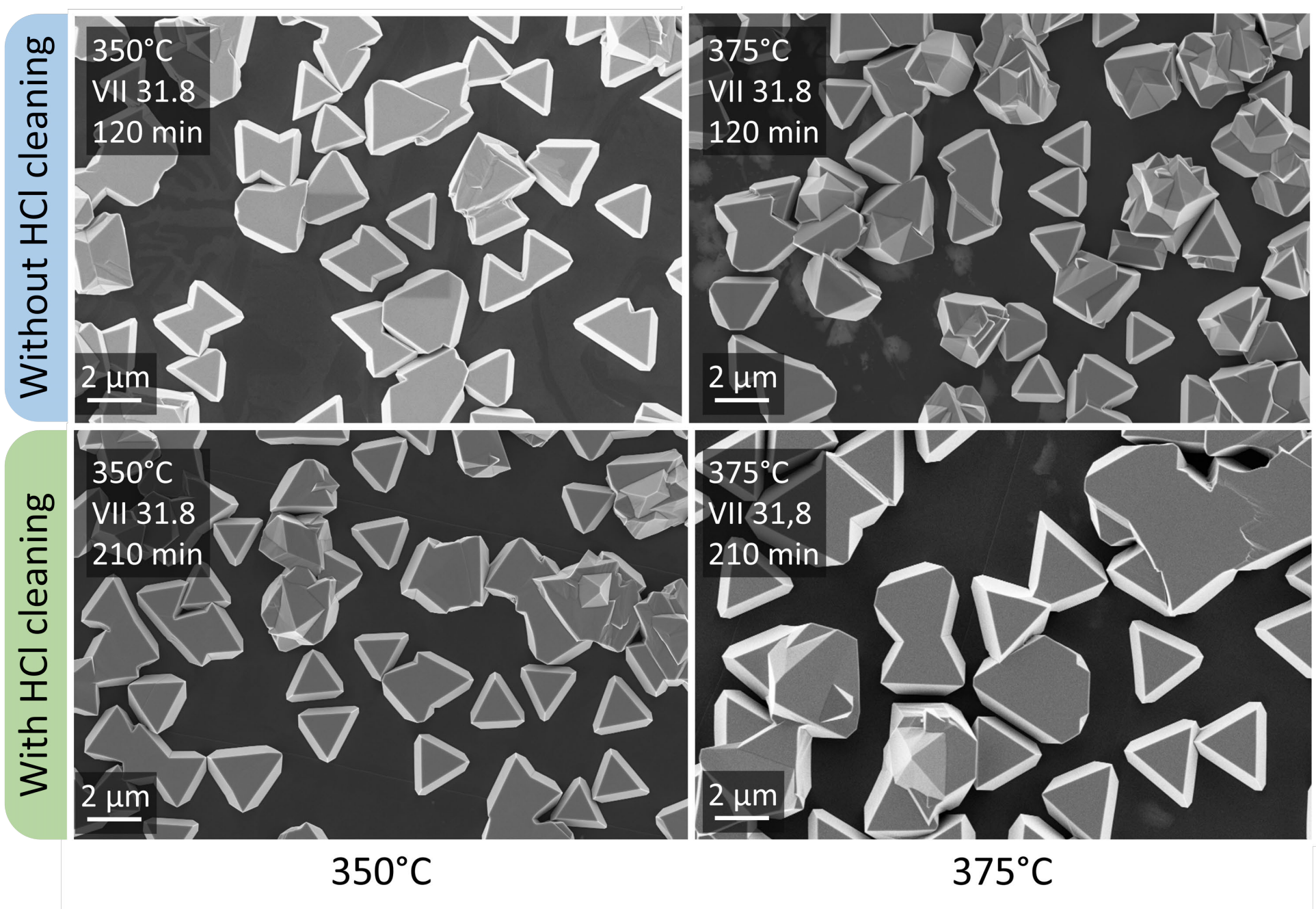

Figure S4: MOVPE growth outcome of Zn_3P_2 on the commercially available epitaxial graphene on Si-face SiC obtained via high-temperature annealing from Graphensic.

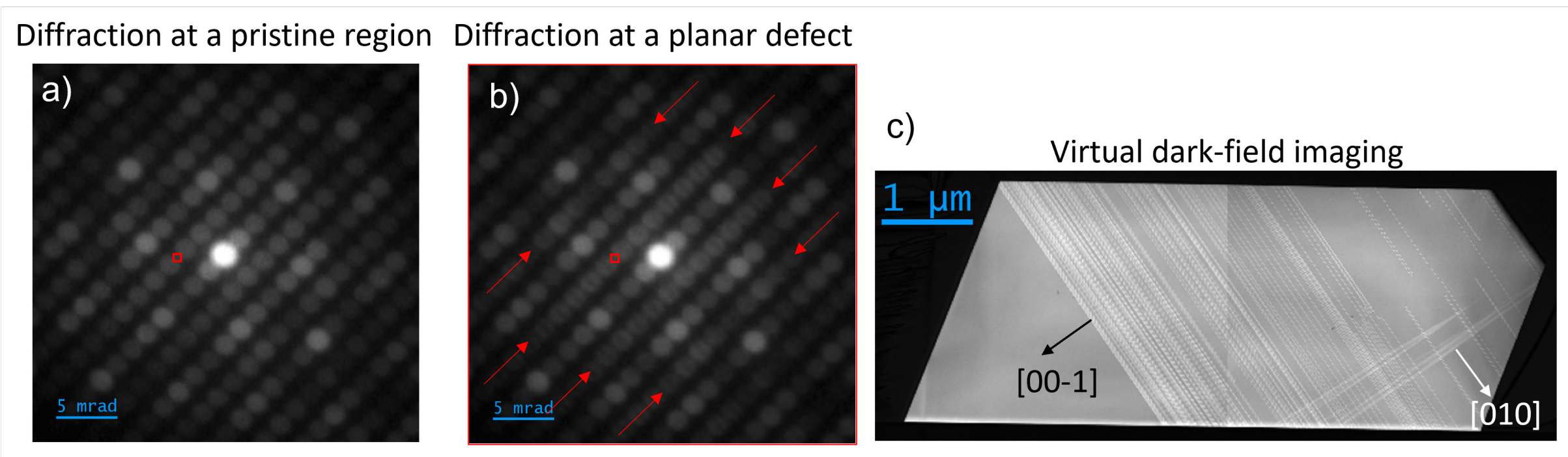

Figure S5: 4D-STEM measurements showing the diffraction patterns for pristine Zn3P2 (a) and Zn3P2 containing an APB (b). The APB perturbs the Bragg intensities of the systematic rows indicated by the red arrows, corresponding to reflections of type (0 j k) with j = 2n + 1 and k an integer. Thus, the virtual aperture drawn as a red square in the diffraction patterns is used to generate high contrast of the APB in the virtual dark-field image shown in (c).

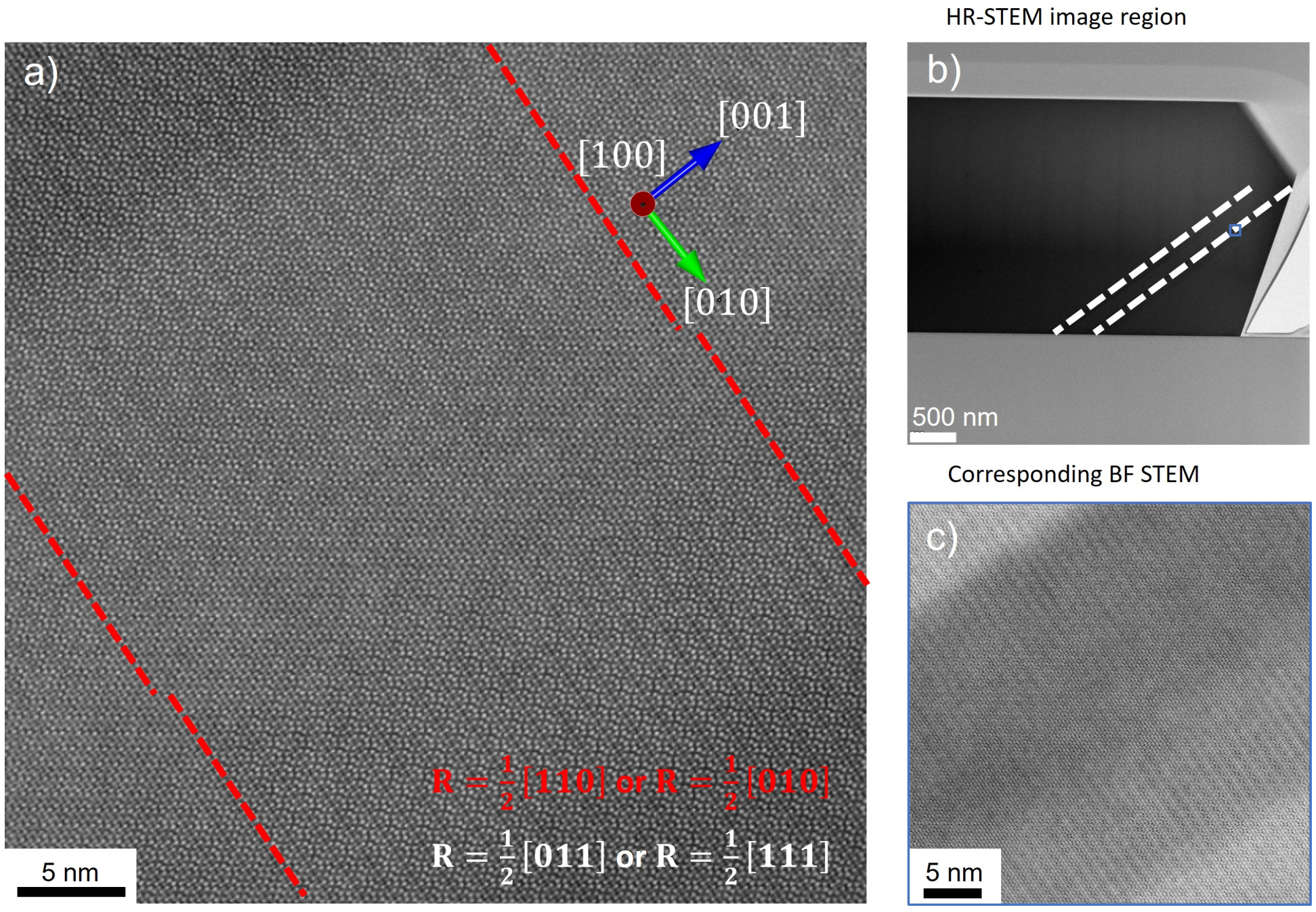

Figure S6: (a) Atomic-resolution HAADF-STEM image showing two (001) ABPs indicated in red intersecting a (110) ABP with R=1/2[011]. The (110) ABP are indicated in white in the BF STEM image (b). The (110) ABP is not on-edge relative to the observation axis, as easily visible on the atomic-resolution BF STEM image (c).

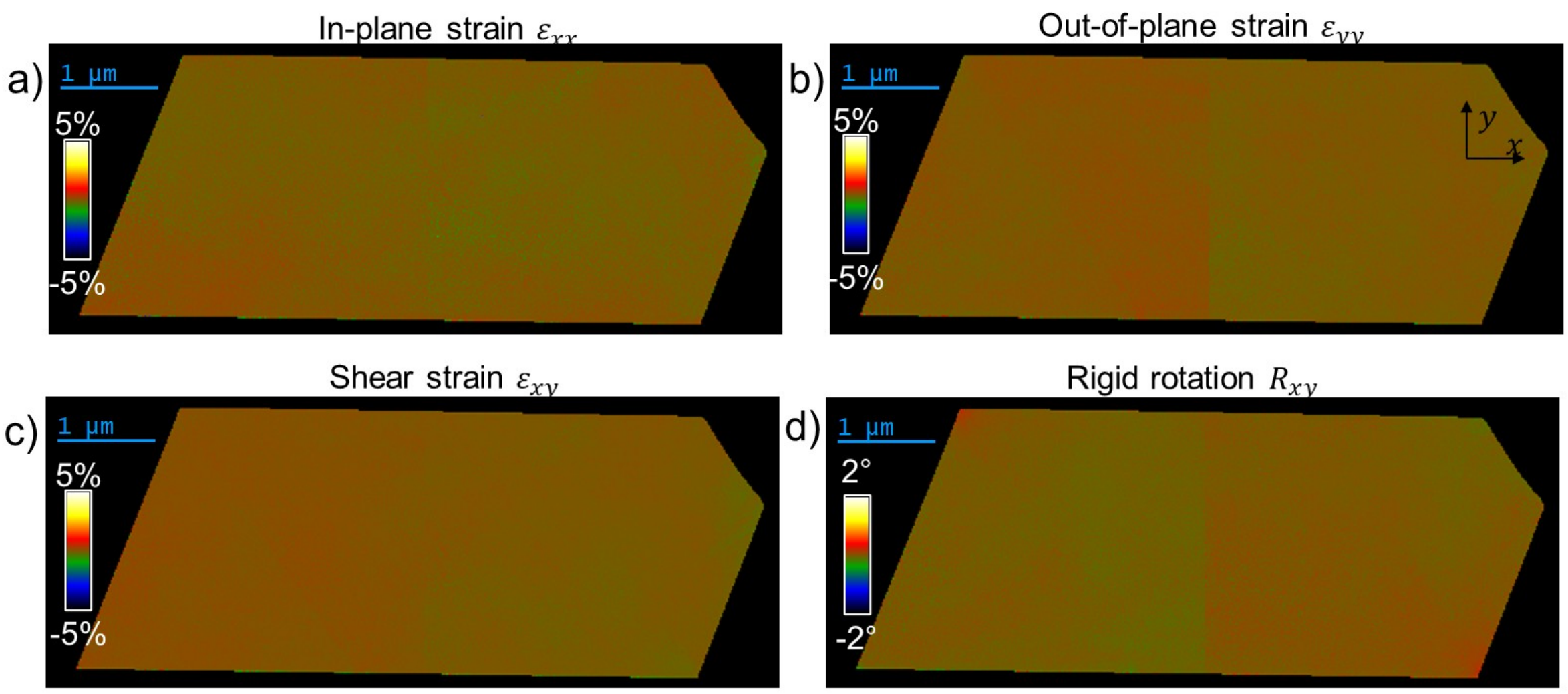

Figure S7: Precession 4D-STEM analysis of strain and lattice rotation in Zn_3P_2. The components of the 2D strain tensor and the rigid rotation are plotted with the x-axis in-plane and the y-axis out-of-plane, as shown in (a-c). No significant strain is observed in the whole crystal grain, with strain variations below ±0.1%. (d) Corresponding lattice rotation map, indicating no perceptible rigid body rotation.

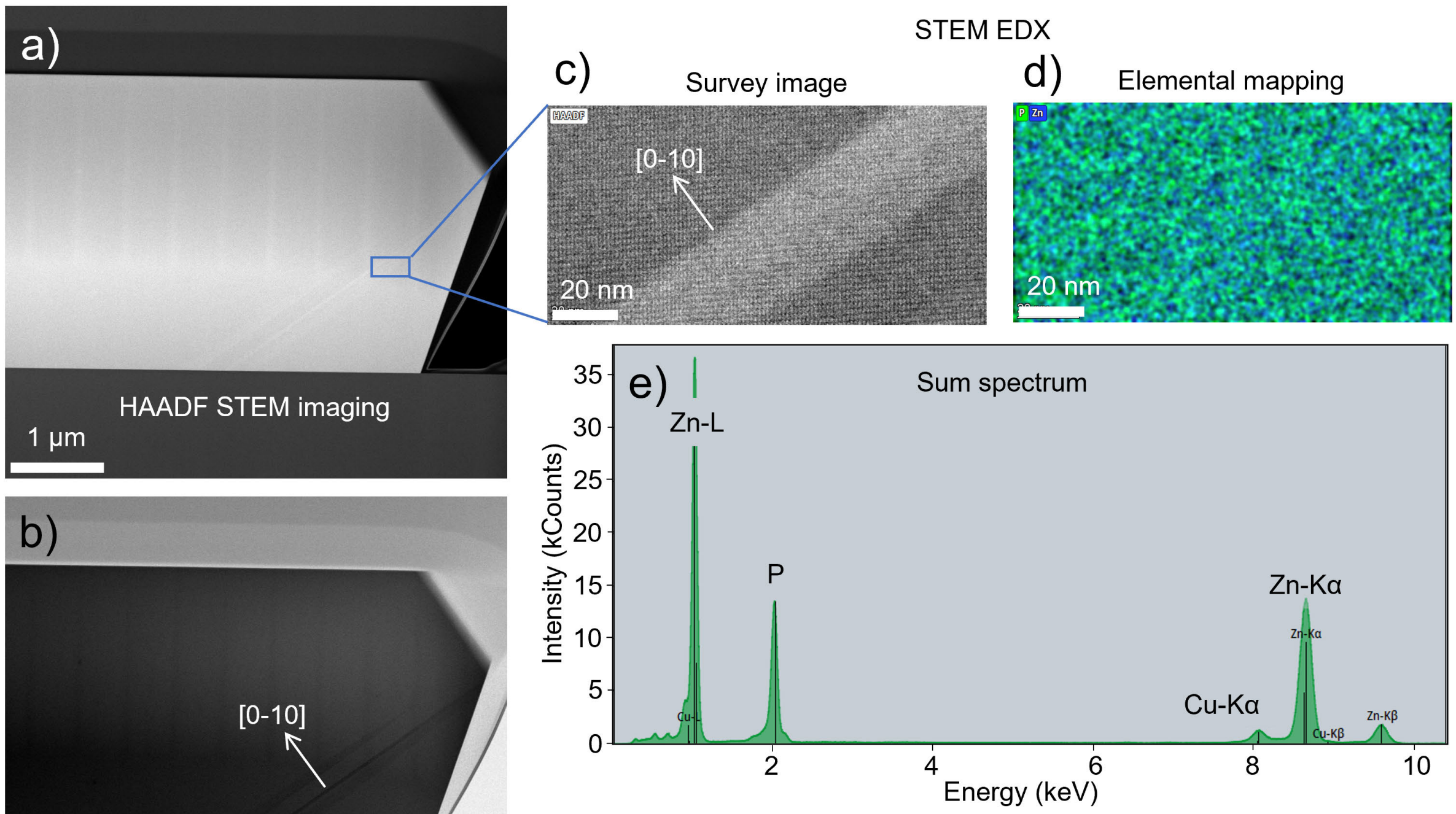

f) Element quantification

Z	Element	Family	Atomic Fraction (%)	Atomic Error (%)
15	P	K	39.51	3.20
30	Zn	K	60.49	3.20

Figure S8: Summary of the STEM-EDX measurements. (a) shows the HAADF and (b) BF STEM images. (c-f) show the elemental mapping of Zn and P at the position of a (110) APB visible on the survey image. A homogeneous composition is observed. The sum spectrum is quantified, and the stoichiometric composition is measured.

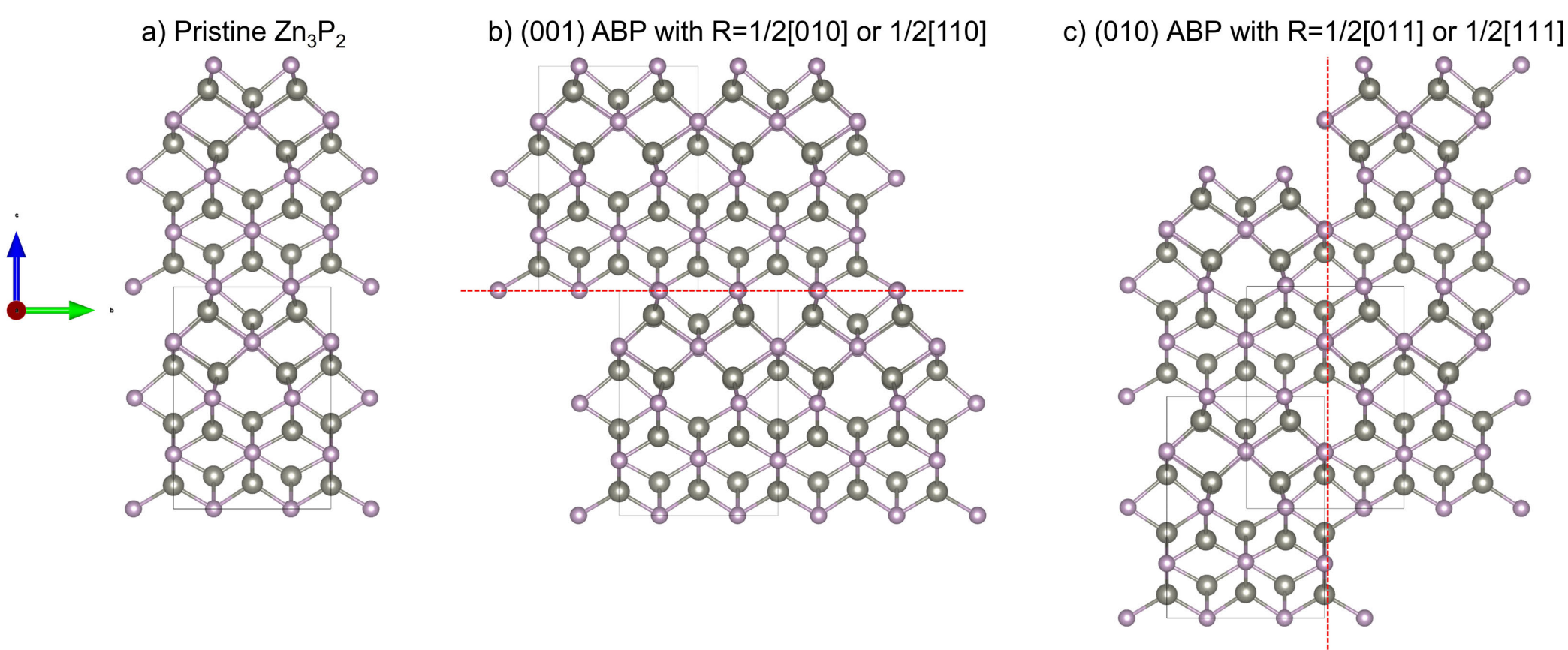

[h]

Figure S9: Zn_3P_2 p4nmc crystal structure displayed using Vesta software of pristine crystal (a) of APB (001). ABP with displacement vector R=1/2[010] or 1/2[110] (b) and of APB with R=1/2[011] or by R=1/2[111] (c).

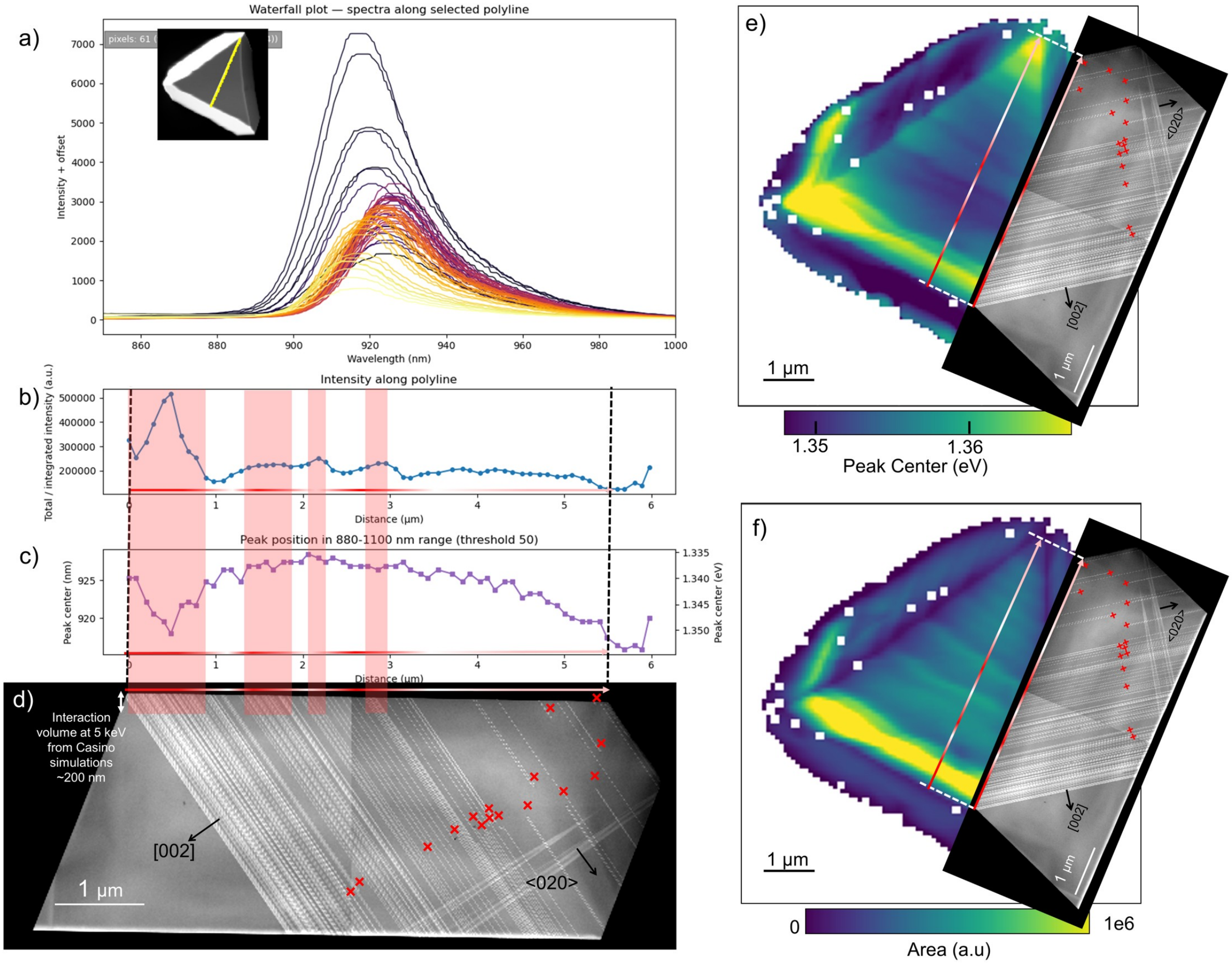

Figure S10: Correlation between cathodoluminescence (CL) emission and antiphase boundary (APB) distribution in a Zn_3P_2 grain. CL measurements were acquired at 10 K, 5 keV and 0.5 nA. (a) Waterfall plot of CL spectra acquired along the selected polyline indicated in the inset, positioned at the same location as the TEM lamella extraction. (b) Integrated CL intensity and (c) peak centre position extracted along the same line, showing spatial variations across the grain. Shaded regions highlight areas of increased APB density. (d) Virtual dark-field image extracted from precession 4D-STEM data, indicating the distribution of APBs within the lamella and interaction volume based on Casino simulations.[1] (e) Spatial map of the CL peak centre energy and (f) integrated emission intensity, overlaid with the TEM lamella. A clear spatial correlation is observed between regions of high APB density and variations in both emission intensity and peak energy. While the correspondence is not one-to-one, the CL variations consistently follow the overall distribution of APBs and their local density, indicating a strong, albeit not perfect, relationship between structural defects and optical response.

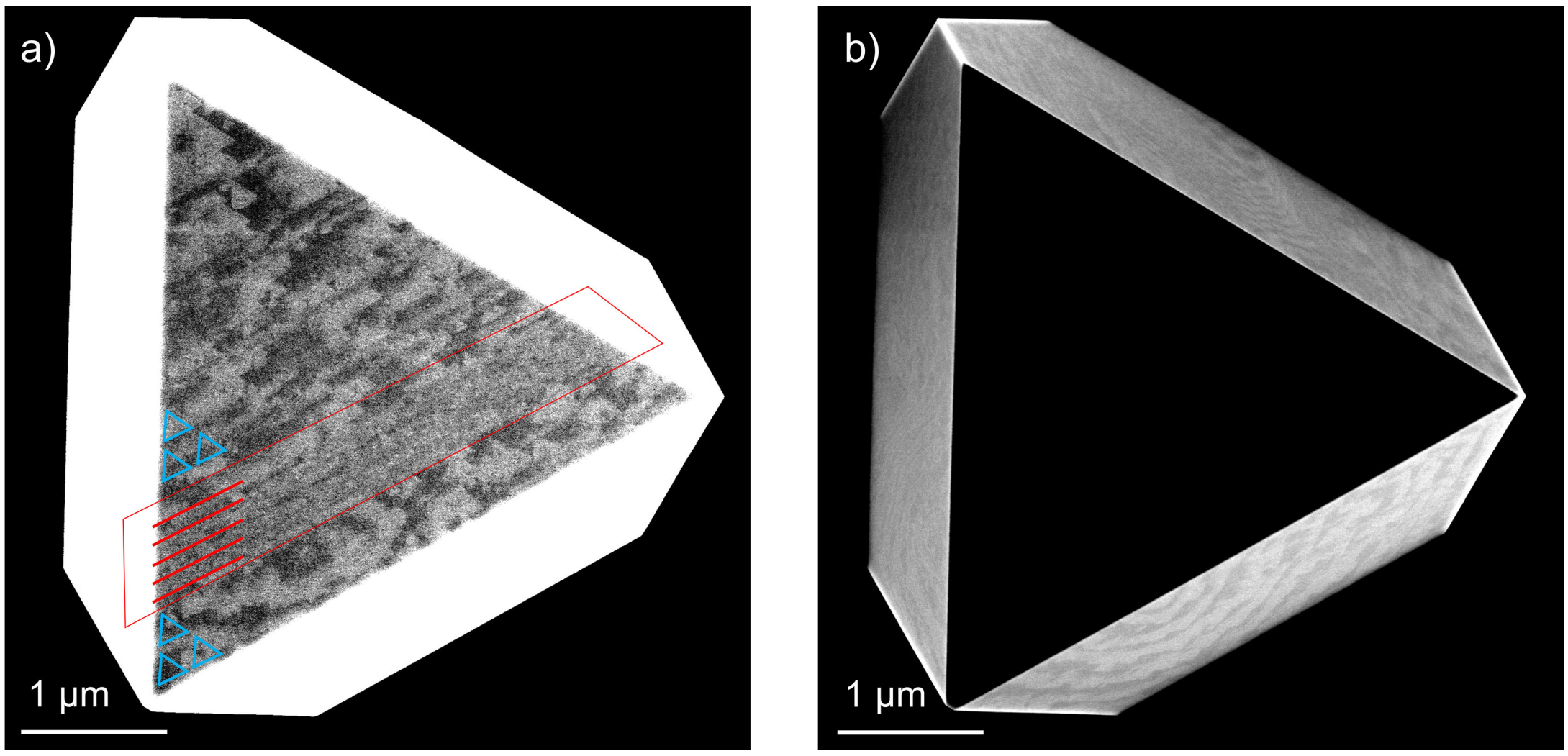

Figure S11: Top-view SEM image of a monocrystalline Zn_3P_2 grain. a) and b) show different contrast filtered images to expose structural variation at the surfaces. The red-marked region in (a) exhibits line-like surface texture, different from the rest of the top surface. Although this grain is different from the one analysed by TEM, it is possible that the variation in surface texture is induced by a high density of APBs.

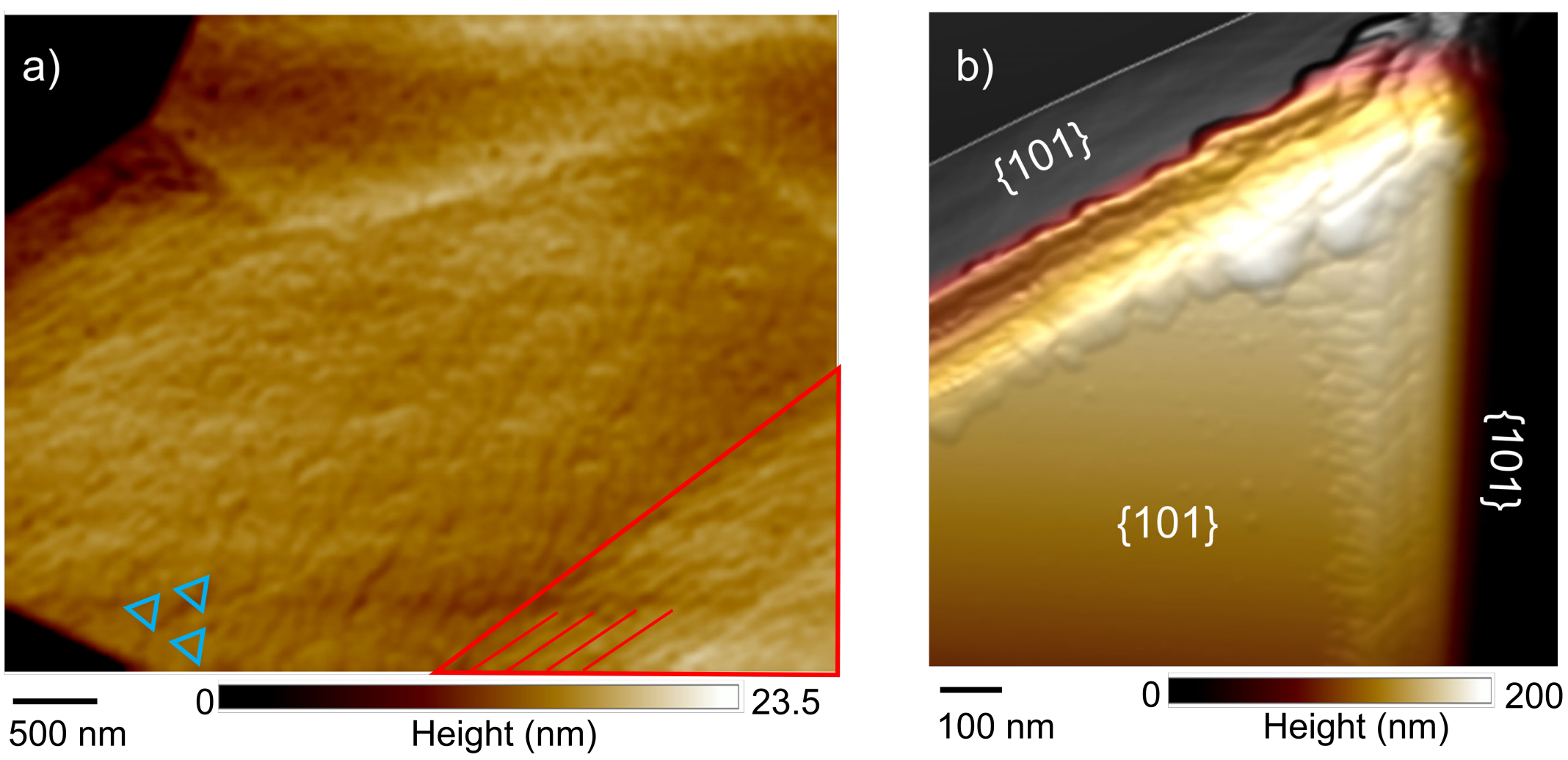

Figure S12: Atomic force micrograph images of Zn_3P_2 grown by MOVPE. a) and b) show different regions of grains. The red marked region in (a) exhibits line-like surface texture, similar to the one observed in Fig. S11. b) shows the topography of the edges where the 101 planes merge together.

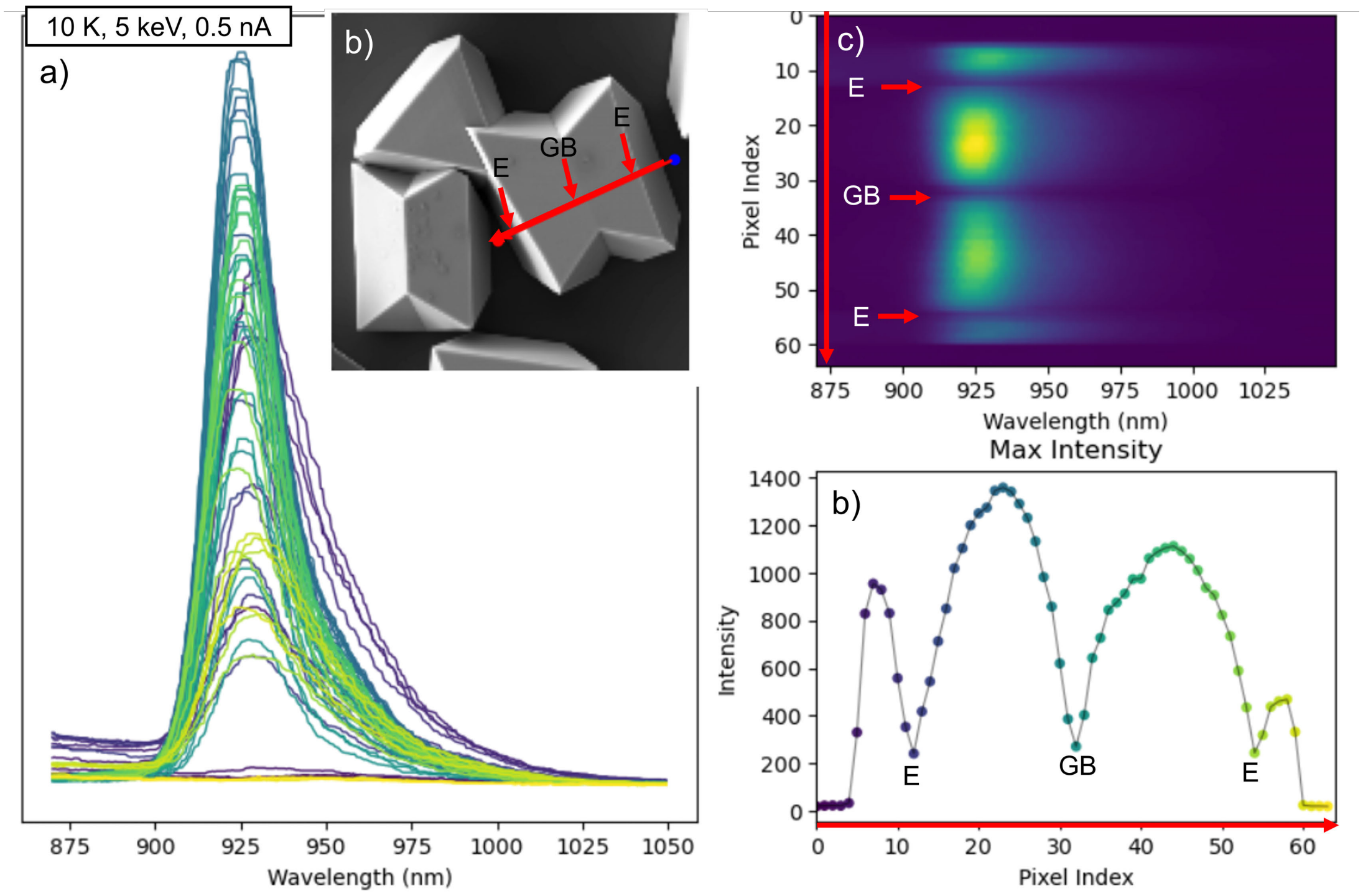

Figure S13: CL measurement of Zn_3P_2 grown by MOVPE. a) shows the emission at 10 K along the indicated line in the SEM image in (b). c) shows the spectra of each pixel along the line in a heatmap, with the grain boundary (GB) and edges (E) indicated. d) shows the maximum intensity of the spectrum at each pixel. A clear quenching of the signal is observed at the GB and the edge, which may be related to the rough surface texture at the edge, observed in the AFM images in Fig. S12.

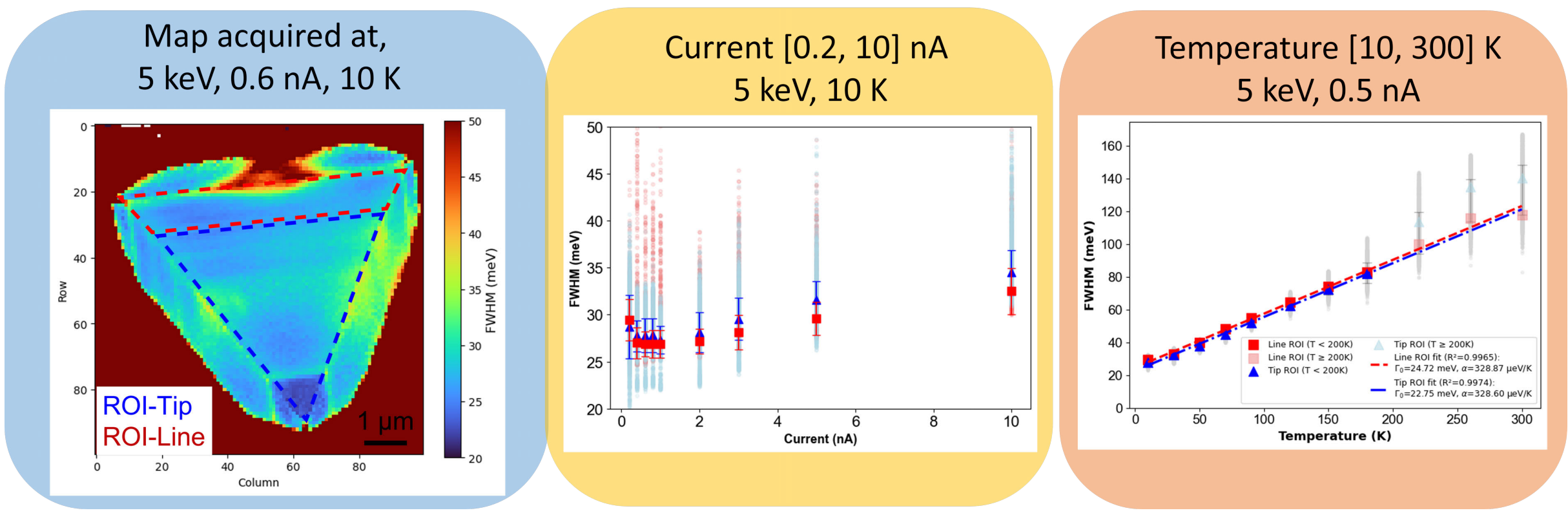

Figure S14: Linewidth corresponding to the fits represented in Fig.**??**. a) Representative FWHM map of the acquisition at 10 K, 5 keV, 0.6 meV. b) FWHM evolution with current and c) with temperature. No significant difference observed between the two ROIs.

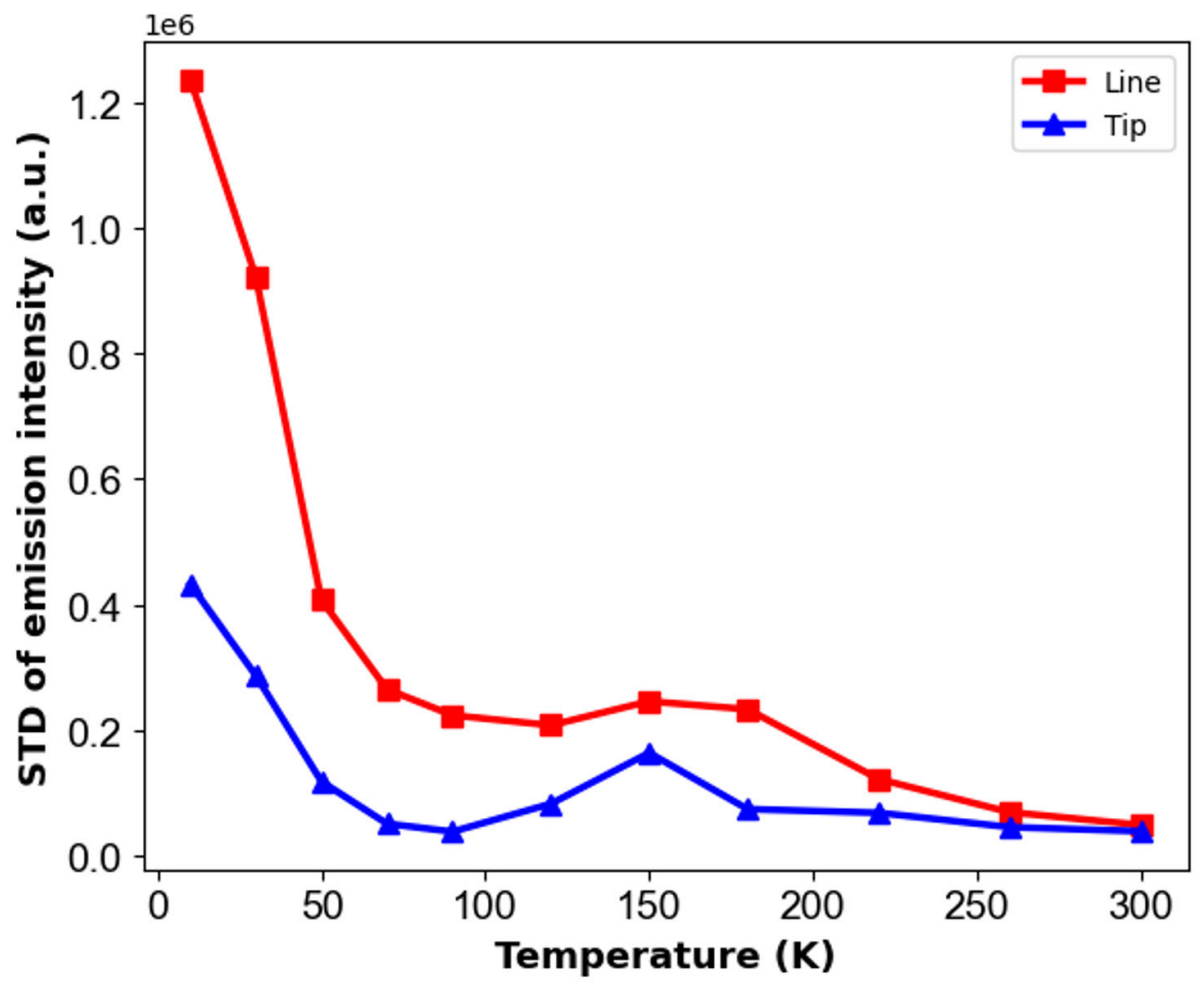

Figure S15: Standard deviation of emission intensity within the two ROIs. A high STD is observed between the different pixel at low T. As the temperature increases, the emission homogenises and the two ROIs become more similar.

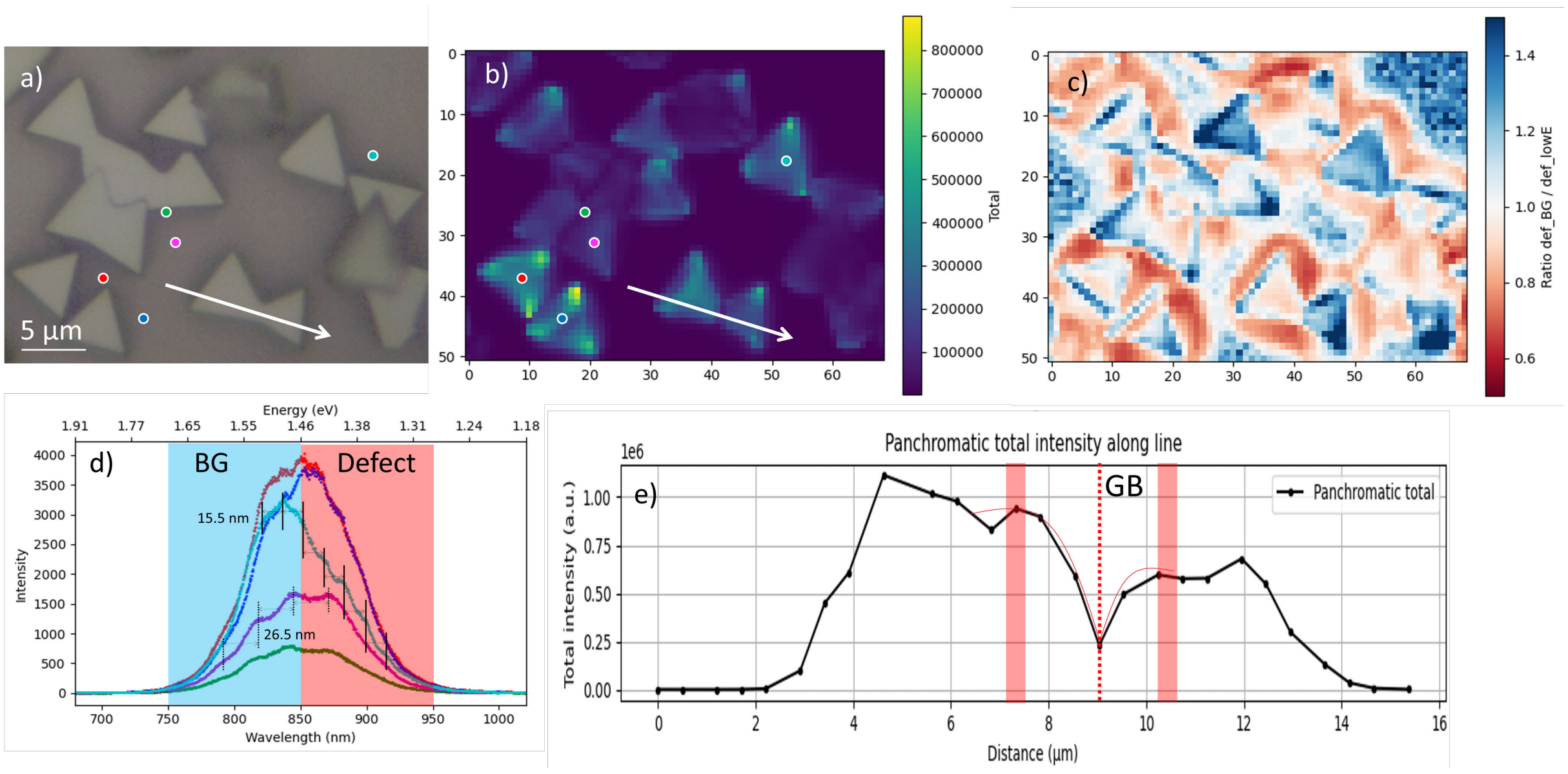

Figure S16: RT-PL map of MOVPE-grown Zn_3P_2 acquired with a 532 nm laser, laser power of 180 μW, 300 l/mm grating and a microscopy objective with NA=0.85. Map contains 50x68 pixel. a) Microscopy image of the mapped zone b) Panchromatic intensity map c) Ratio of the integrated intensity [750-850] nm in blue and [850-950] nm in red. More red appearing regions do have a higher amount of sub-bandgap emission. Large variations in grain colouring are observed, also resembling lines as observed in CL. d) Example spectra at the indicated positions in (a), with the noted integrated ranges in red and blue. Interference patterns are visible in the spectrum, which do, however, not match any dimension of the grain, and therefore remain of unclear origin. e) Panchromatic intensity linescan across a grain boundary, indicated in (b), pointing towards a recovery length in the order of the micrometre.

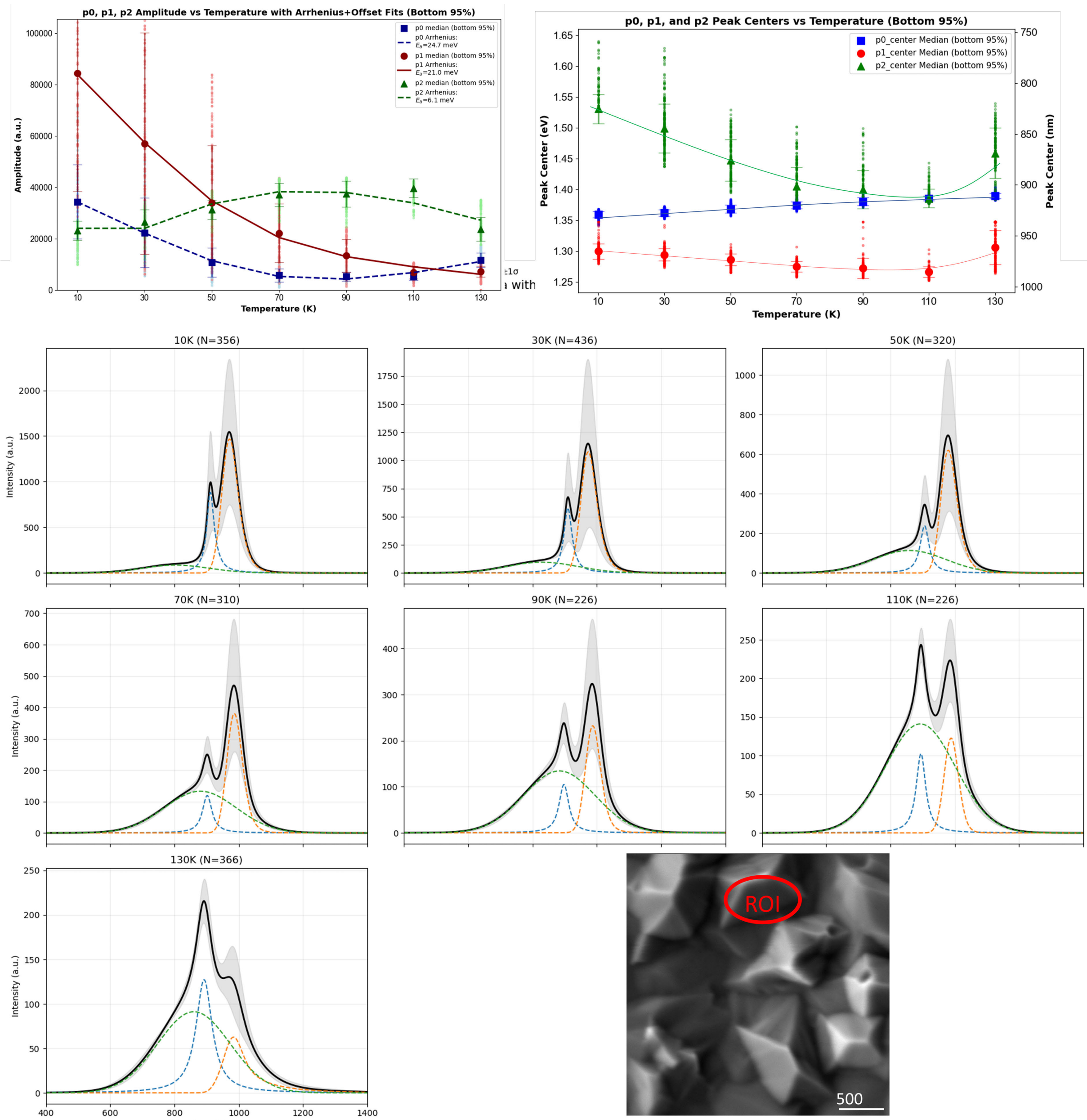

Figure S17: CL emission behaviour of the MBE-grown Zn_3P_2 thin film with varying temperature. Arrhenius plot, peak centre evolution and the average spectrum in the ROI at each temperature, with the average fit component p0 (HE),p1 (LE) and p2. A broad Gaussian (green) was fitted above the HE emission (blue), which redshifts with temperature and is broader than the other emissions, likely due to band-to-band or tail-to-tail transitions. The p1 (LE) emission behaves similar with temperature as the reported emission in the MOVPE-grown sample.

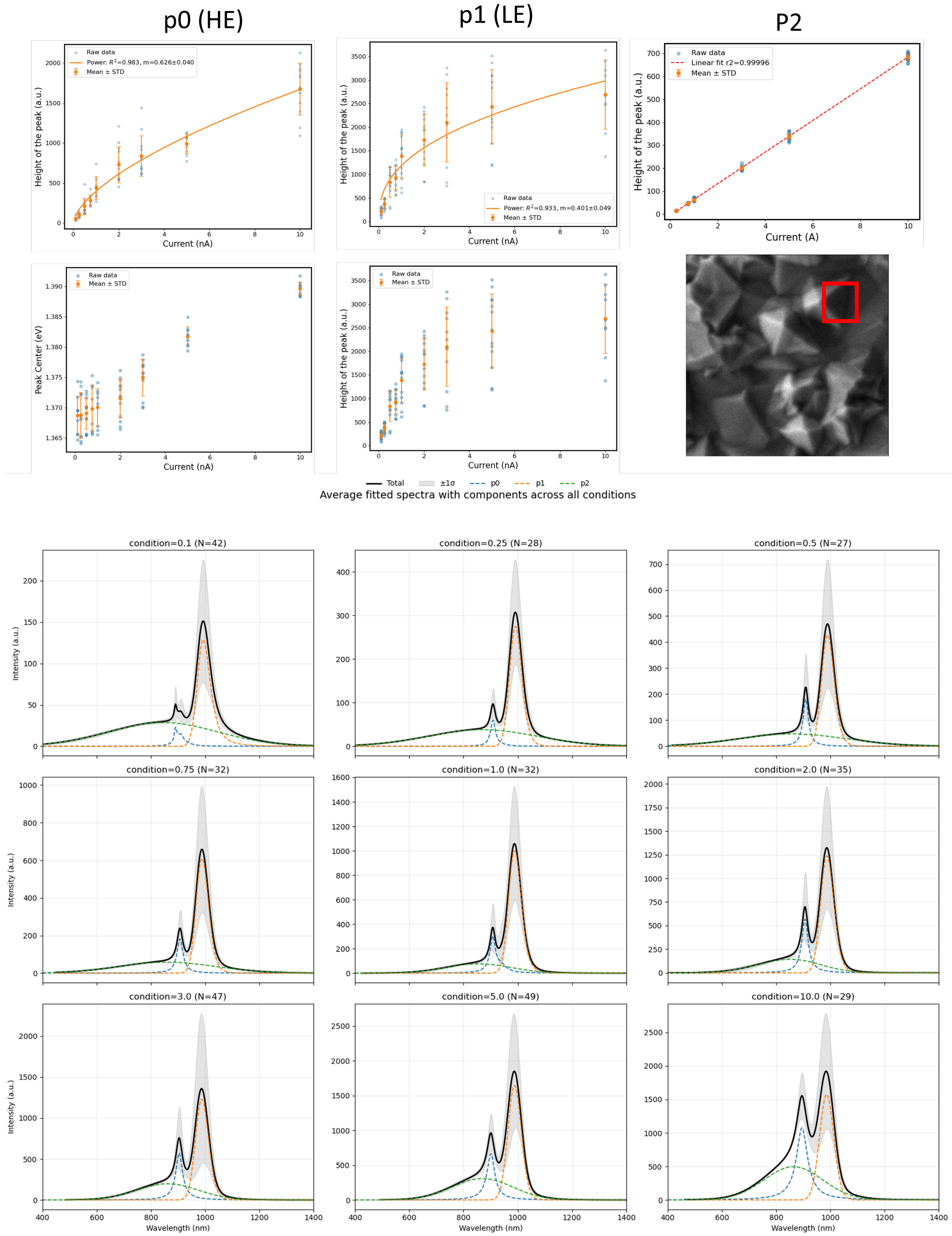

Figure S18: CL emission behaviour of the MBE-grown Zn_3P_2 thin film with varying excitation current. For all three peaks, the amplitude evolution is given, indicating saturation for the HE and LE peaks and a linear behaviour for the p2 peak, further supporting bandgap-related emission of p2. p1(LE) behaves similar with current as the reported emission in the MOVPE-grown sample.

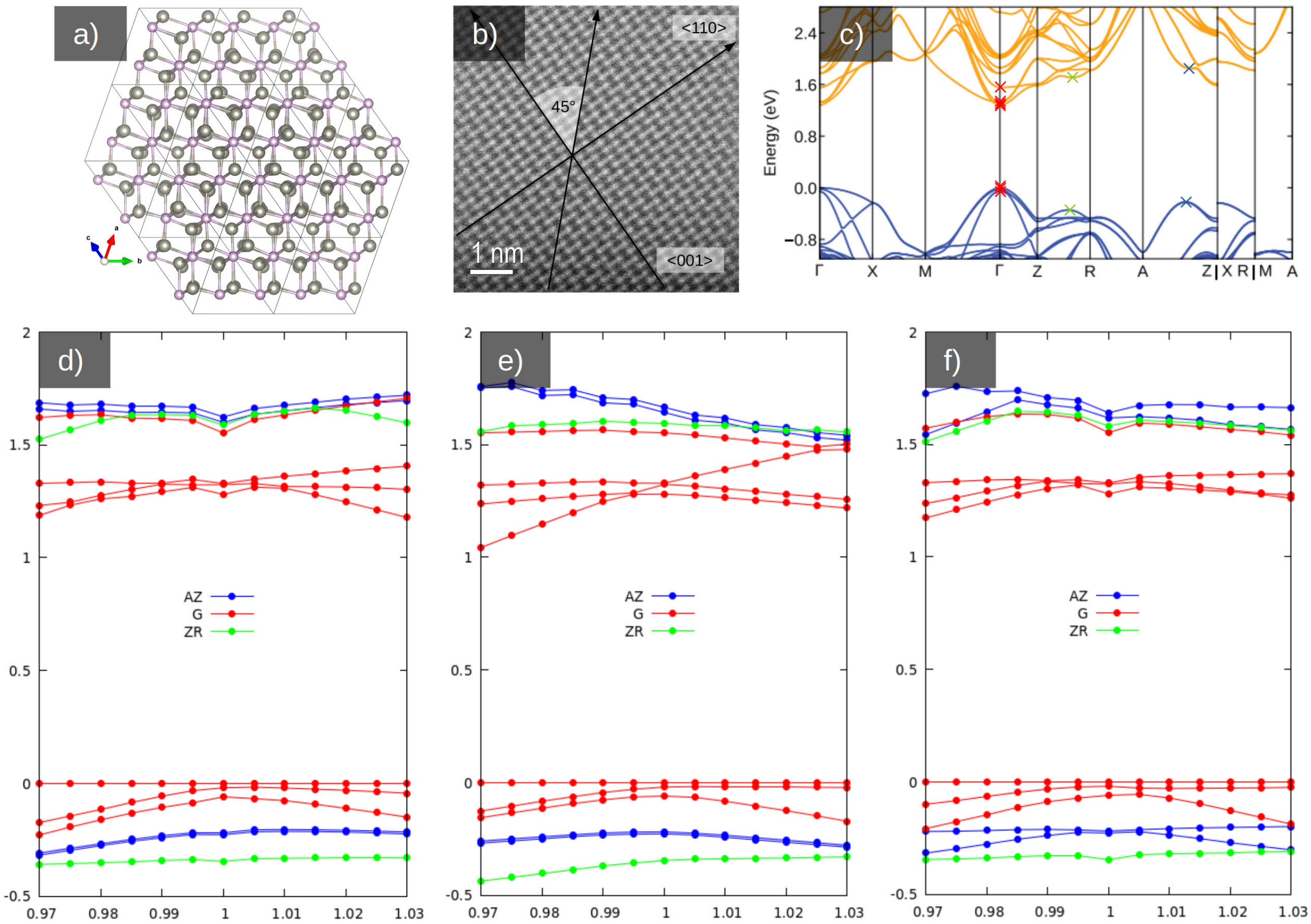

Figure S19: Density functional theory (DFT) analysis of strain effects in Zn_3P_2. (a) Model crystal structure projected along [$\bar{1}$11] with the <101> vector pointing upwards. (b) High-resolution TEM image in matching projection and orientation. The three indexed directions illustrate the three directions in which uniaxial strain was applied. (c) Calculated electronic band structure along high-symmetry directions. Crosses mark electronic states whose evolution under strain was investigated. (d–f) Evolution of selected electronic states under uniaxial strain applied along (d) <001>, (e) 45° between <001> and <110>, and (f) <110> directions. The energy shifts of valence and conduction band states reveal a pronounced anisotropy in the strain response, indicating direction-dependent modifications of the band edges and suggesting that the electronic structure is sensitive to local lattice distortions.

References

(1) Hagger, T.; Freitas, H. R.; Mastropasqua, C.; El Alouani, A.; Marinoni, S.; Kawashima, N.; Lemerle, R.; Wodzislawski, K. A.; Dede, D.; Botti, S., et al. Link between graphene features and the resulting functionality of quasi-van der Waals Zn_3P_2. *CrystEngComm* **2026**, *28*, 635–648.
